# Integrated miRNA-mRNA analysis reveals the roles of miRNAs in the replanting benefit of *Achyranthes bidentata* roots

**DOI:** 10.1038/s41598-021-81277-6

**Published:** 2021-01-15

**Authors:** Yan Hui Yang, Ming Jie Li, Yan Jie Yi, Rui Fang Li, Cui Xiang Li, Heng Yang, Jing Wang, Jing Xuan Zhou, Sui Shang, Zhong Yi Zhang

**Affiliations:** 1 College of Bioengineering, Henan University of Technology, Lianhua Street 100, High-technology Zero, Zhengzhou, 450001 Henan Province China; 2 College of Crop Sciences, Fujian Agriculture and Forestry University, Jinshan Road, Cangshan District, Fuzhou, 350002 China

**Keywords:** miRNAs, Plant molecular biology

## Abstract

The yield and quality of the medicinal plant *Achyranthes bidentata* can be increased when it is replanted into a field cultivated previously with the same crop, however, fundamental aspects of its biology (so-called “replanting benefit”) still remain to be elucidated. miRNAs are sRNA molecules involved in the post-transcriptional regulation of gene expression in plant biological processes. Here, 267 conserved and 36 novel miRNAs were identified in *A. bidentata* roots. We compared the miRNA content of the roots (R1) from first-year planting with that of the roots (R2) of second-year replanting, and screened 21 differentially expressed (DE) miRNAs. Based on in silico functional analysis, integrated miRNA-mRNA datasets allowed the identification of 10 miRNA-target family modules, which might participate in the benefit. The expression profiles of the miRNA-target modules were potentially correlated with the presence of the replanting benefit. The indication was that the miRNA-responsive continuous monoculture could reprogram miRNA-mRNA expression patterns, which possibly promote the root growth and development, enhance its transport activity and strengthen its tolerance to various stresses, thereby improving *A. bidentata* productivity as observed in the replanting benefit. Our study provides basic data for further research on the molecular mechanisms of the benefit in *A. bidentata*.

## Introduction

For a number of crop species, replanting or continuous monoculture into a field previously cultivated with the same crop results in a yield penalty^1–4^. In some cases, however, the effect is the opposite, a phenomenon known as the replanting benefit^5–7^. A typical example of the latter phenomenon is that of the perennial herbaceous species *Achyranthes bidentata* (Amaranthaceae)^6,7^, a species cultivated for the medicinal properties thought to be present in the extracts of its dried roots, an ingredient widely used in traditional Chinese medicine^8,9^. The factors responsible for this replanting benefit are generally assumed to be associated with either unknown component(s) of its root exudate, changes in the nutritional environment and/or the effect of the first crop on the rhizosphere microflora^5,7^. The sustainable and profitable cultivation of *A. bidentata* requires sufficient understanding of the mechanistic basis of the replanting benefit. Although our previous study disclosed preliminary data on the molecular regulation of its mRNA transcription^6^, the way in which replanting *A. bidentata* causes the associated post-transcriptional changes and regulation remains unknown.

Recently, the post-transcriptional regulation of genes by a group of small RNAs (sRNAs), including microRNAs (miRNAs) and small interfering RNAs (siRNAs), has been revealed to be a new mechanism in plant growth and development, signal transduction, protein degradation and responses to environmental stimuli^10–14^. Of these sRNAs, miRNAs are endogenous 20–24 nt non-coding RNAs, processed from RNA precursors that are capable of forming imperfectly complementary hairpin structures through the DICER-LIKE-1 (DCL1) enzyme^13,14^. They are known to affect the level of gene expression by binding to their target messenger RNAs (mRNAs through complementary base-pair interactions and inducing their degradation or repressing their translation in organisms^11,14,15^. As their sequences are so highly conserved across the eukaryotes, they are believed to represent an evolutionarily ancient component of gene regulation. To date, numerous studies have demonstrated that miRNA-mediated gene regulation plays an important role in various environmental responses of several plant species, including *Arabidopsis thaliana*^14^, *Populus*^15^, wheat^16^ and ramie^17^.

The hypothesis in the present study was that miRNA activity may underlie some at least part of the regulatory mechanism associated with the replanting benefit of *A. bidentata*. High-throughput RNA sequencing (RNA-seq) technology is a powerful tool that allows concomitant sequencing of millions of sRNAs from a single tissue^10,16,17^. To date, numerous miRNAs involved in plant environmental responses have been revealed via this technology. In order to obtain a global picture of the miRNA content of *A. bidentata* and better decipher any miRNA activity involved in regulating the replanting benefit, we chose to investigate specifically the *A. bidentata* roots (the medicinal part), which are also the most sensitive tissue to the replanting gain and used in the experiments^6^. We performed sRNAome sequencing using the RNA-seq platform and compared the miRNAomes of the roots of the first-year planting (R1) and the second-year replanting (R2) in order to explore the global miRNA changes. In previous research^6^, we obtained a set of *A. bidentata* transcriptome data using the RNA-seq platform, compared the transcriptome of the roots of R1 (i.e. normal growth (NG)) and R2 (i.e. continuous monoculture (CM)), and preliminarily identified as candidate genes involved in the replanting benefit. To investigate miRNA-target modules participated in the benefit, an analysis of integrated differentially expressed (DE) miRNA and mRNA ‘omic’ datasets allowed the identification and functional analysis of the miRNA-target modules, disclosing the miRNA roles potentially responsible for the replanting benefit.

## Results

### Comparison of morphological characteristics and biomass between R1 and R2 roots

**Figure 1:**
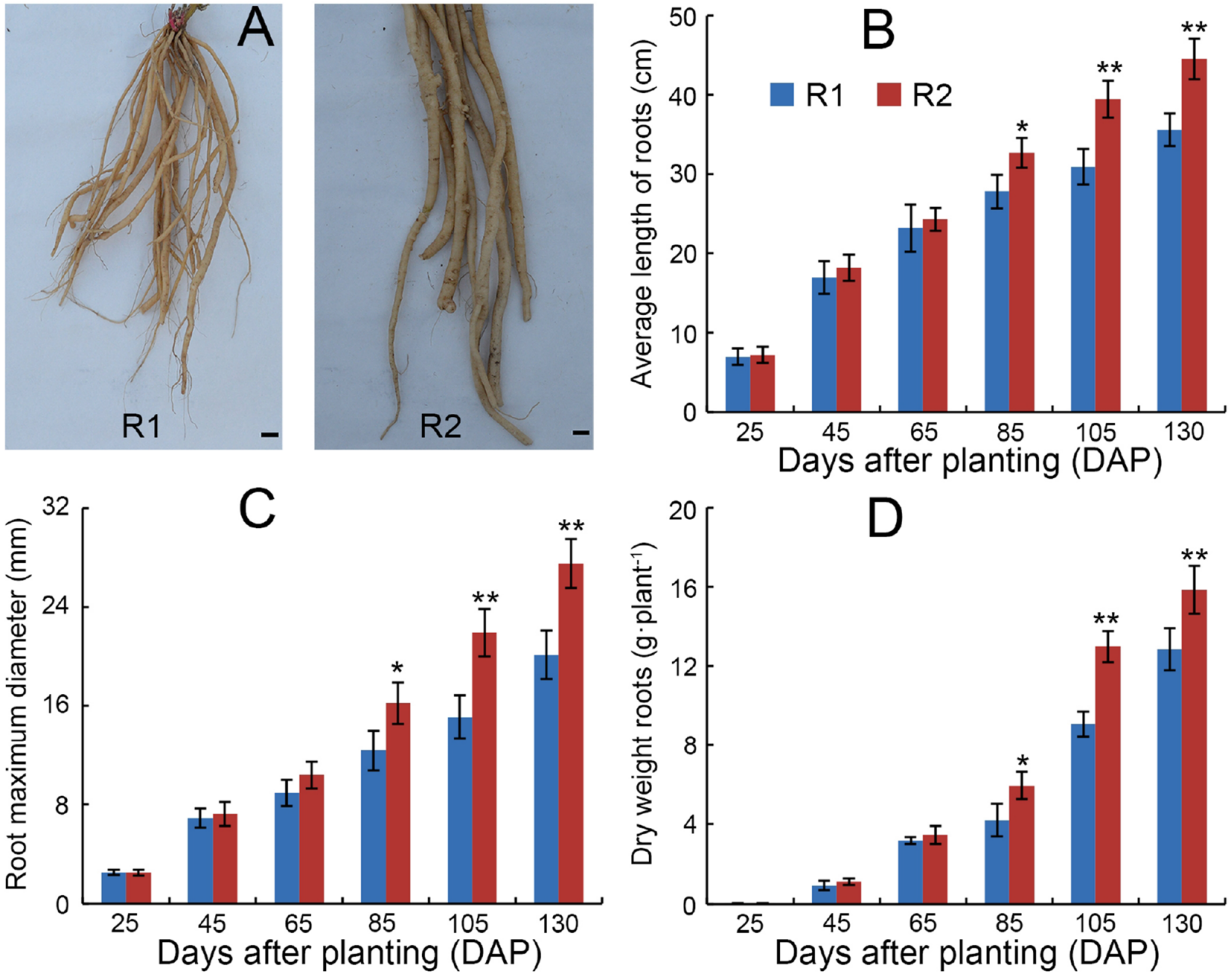
The morphological characteristics from R1 and R2 roots. (**A**) The root photograph at the 130 DAP (bar = 2 cm), (**B**,**C**) root morphological index and (**D**) biomass. The bars represent the standard error (*n* = 3); “*” and “**” represent different significance P < 0.05 and 0.01, respectively. The same below.

The exposure of *A. bidentata* to the continuous monoculture condition resulted in significant promotion of the root growth and development, as indicated by the favorable root phenotype in R2 compared with R1 roots (Fig. 1A). For example, the average length of R2 roots was longer than that of R1 at most of the sampling time points (especially significant from 85 to 130 days after planting (DAP)) (Fig. 1B). Similarly, the root maximum diameter from R2 exhibited significantly wider than that of R1 from 85 to 130 DAP (Fig. 1C). Consistent with the root length and width increase, the root biomass (dry weight) from R2 plants between 85 and 130 DAP was significantly higher than that in R1 (Fig. 1D). Based on these morphological changes in replanted *A. bidentata* roots, after approximately 85 DAP, the significant characteristic of the replanting benefit by these indexes was obviously observed. To explore important miRNA regulation information of the replanting benefit, we chose the 85 DAP *A. bidentata* roots as experimental materials for analyzing the changes of R1 and R2 miRNA-mRNA modules.

### Identification of conserved miRNAs

**Figure 2:**
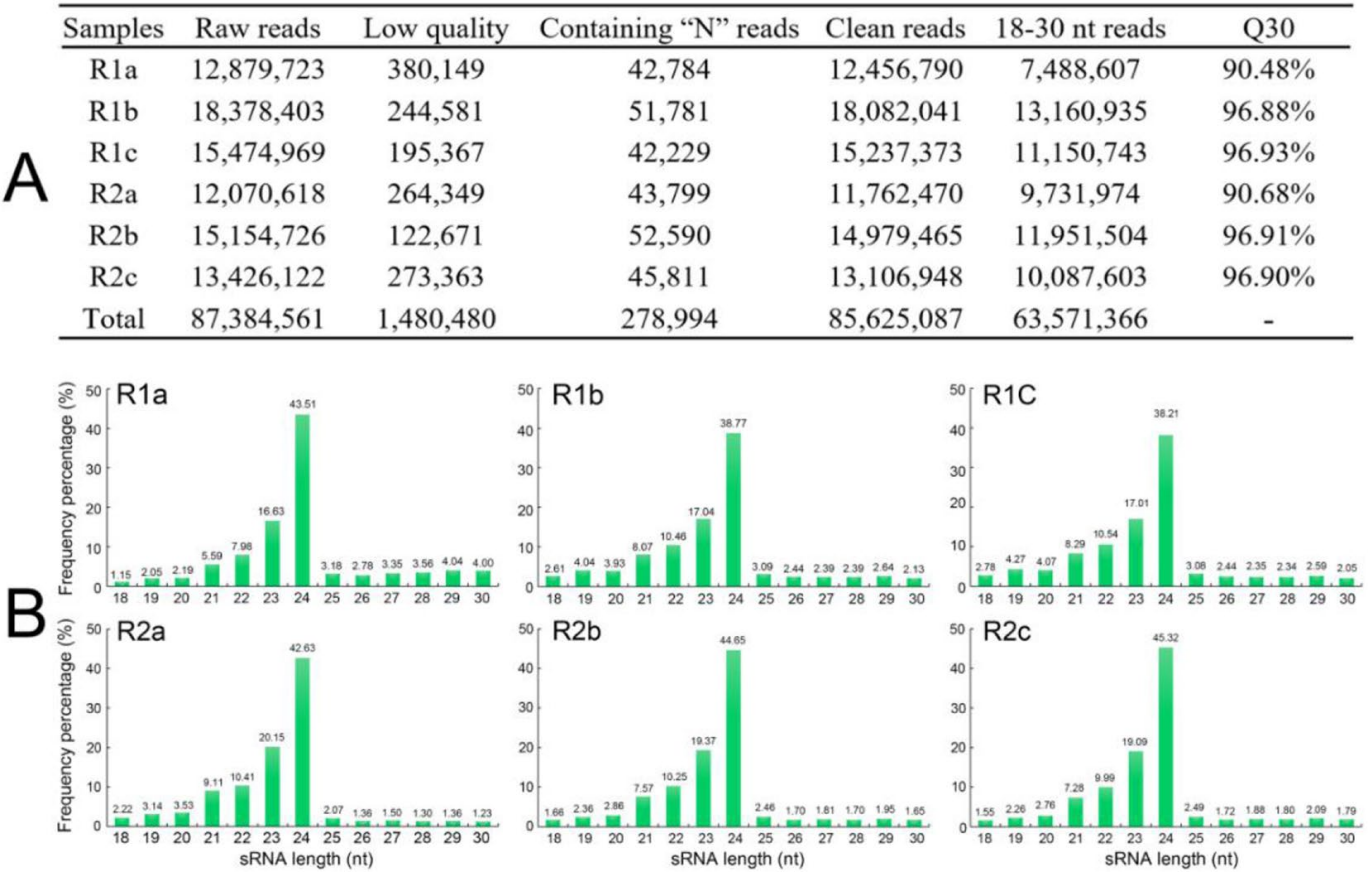
Summary statistics of sRNAs (**A**) and length distribution of clean reads (**B**) between R1 and R2 samples.

To identify miRNAs in *A. bidentata*, the six sRNA libraries from R1 and R2 roots at 85 DAP with three biological replicates were generated and analyzed by RNA-seq technology. Data from the sequencing results are summarized in Fig. 2A. All raw reads was deposited at the NCBI Sequence Read Archive (SRA, http://www.ncbi.nlm.nih.gov/sra) under the accession number PRJNA564403. For each individual sample, the number of retrieved raw reads ranged between 12.1 million and 18.4 million, presenting an extensive resource for the discovery of miRNAs. After filtering out the low-quality reads and those containing “N” reads, the number of remaining clean reads ranged between 11.8 and 18.1 million. The Q30 value of the raw reads was > 90% for all samples. For further sRNA analysis, a total of 63,571,366 sequences ranging 18–30 nt in length were extracted from all the six libraries. Overall, > 76% of the clean reads were in the range 21–24 nt in length, the majority of which were 24 nt in size, followed by 23 nt, 22 nt and 21 nt, all in the six libraries of *A. bidentata* (Fig. 2B).

For each sample tested, the 18–30 nt reads were mapped to the *A. bidentata* transcriptome. The resulting proportions of relevant reads were 42.43–49.87% in the R1 samples, and 43.10–46.69% in the R2 samples (Table S1 of Supplementary Material 1), suggesting that the percentage of sRNA-mapped transcriptome of each library was < 50% of the total. These obtained sRNAs were further matched with non-coding RNAs deposited in the public databases, in order to remove non-coding RNAs (ribosomal (r)RNA, transfer (t)RNA, small nuclear (sn)RNA and small nucleolar (sno)RNA), these accounted for 6.90–8.24% of the reads in the R1 samples, and 4.80–6.41% in the R2 samples (Table 1). Following this removal, the remaining sRNAs were compared with the mature miRNAs documented in the miRBase database. Overall, 21,324–55,204 reads in the R1 samples and 36,193–45,843 reads in the R2 samples were matched to a total of 267 miRNAs belonging to 45 miRNA families (34 in R1a, 35 in R1b, 33 in R1c, 36 in R1a, 37 in R1b and 33 in R1c) (Table 2 and Table S2 of Supplementary material 1). Of these miRNA families, 29 included multiple members (e.g. 23 in abi-miR159), whereas 16 included only one member (Table S2 of Supplementary Material 1).

**Table 1:** Category distribution of unique sRNAs mapped to the *A. bidentata* transcriptome.

Category	sRNA number (Percentage %) of each sample					
	R1a	R1b	R1c	R2a	R2b	R2c
Total	3,177,672 (100.00)	6,511,347 (100.00)	5,561,189 (100.00)	4,194,032 (100.00)	5,579,983 (100.00)	4,600,281 (100.00)
**Known ncRNA**	219,234 (6.90)	534,307 (8.21)	457,954 (8.24)	201,171 (4.80)	357,644 (6.41)	278,077 (6.05)
rRNA	210,361 (6.62)	515,592 (7.92)	442,267 (7.95)	196,385 (4.68)	349,506 (6.26)	270,876 (5.89)
tRNA	7 (0.00)	9 (0.00)	6 (0.00)	1 (0.00)	3 (0.00)	6 (0.00)
snRNA	7341 (0.23)	13,756 (0.21)	11,505 (0.21)	3299 (0.08)	5926 (0.11)	5403 (0.12)
snoRNA	1525 (0.05)	4950 (0.08)	4176 (0.08)	1486 (0.04)	2209 (0.04)	1792 (0.04)
Conserved miRNA	21,324 (0.67)	55,204 (0.85)	47,074 (0.85)	45,247 (1.08)	45,843 (0.82)	36,193 (0.79)
Unannotated sRNA	2,937,114 (92.43)	5,921,836 (90.95)	5,056,161 (90.92)	3,947,614 (94.13)	5,176,496 (92.77)	4,286,011 (93.17)

**Table 2:** Summary of conserved miRNAs identified between R1 and R2 samples.

Types	Total	R1a	R1b	R1c	R2a	R2b	R2c
Conserved miRNA families	45	34	35	33	36	37	33
Conserved miRNA members	267	176	196	192	189	194	169
Unique sRNA sequences	3504	462	693	632	584	596	537
Total sRNAs	250,885	21,324	55,204	47,074	45,247	45,843	36,193

In order to assess the characteristics of deeply conserved miRNAs in *A. bidentata*, we aligned the aforementioned 45 miRNA families to the mature plant miRNAs to isolate the highly conserved molecules. We found that 14 abi-miRNA families of *A. bidentata* were highly homologous to miRNAs across more than 20 other plant species, for example, abi-miR396 was identical and conserved in 35 other species. Furthermore, 21 abi-miRNA families were moderately conserved across more than two plant species. However, the remaining 10 abi-miRNA families, the sequences of which were present in only one other species, were poorly conserved. For example, abi-miR8155 was only exactly identical to cpa-miR8155 of *Carica papaya* (Tables S2, S3 of Supplementary Material 1), but this family was not found in other plants. Several miRNA families, such as miR156, miR159 and miR162, have been reported to be deeply conserved in various plant species, and the majority of these conserved miRNAs tend to be abundant^18^. In the 14 highly conserved miRNAs, the most abundant in each library was conservative miR159a (> 11,000 counts), followed by the members of abi-miR162, abi-miR166 and abi-miR319 (Tables S2, S3 of Supplementary Material 1). However, these poorly conserved abi-miRNA families were represented by no more than 20 reads in each library (Table S2 of Supplementary Material 1). The extent of these miRNA family conservation of these families across other plants was demonstrated by an alignment with the mature sequences of 45 other species (Table S3 of Supplementary Material 1), suggesting that the majority of these miRNA families from *A. bidentata* were conserved and identical to those from certain eudicots (e.g. *A. thaliana*, *Glycine max* and *Malus domestica*).

### Identification of novel miRNAs

**Figure 3:**
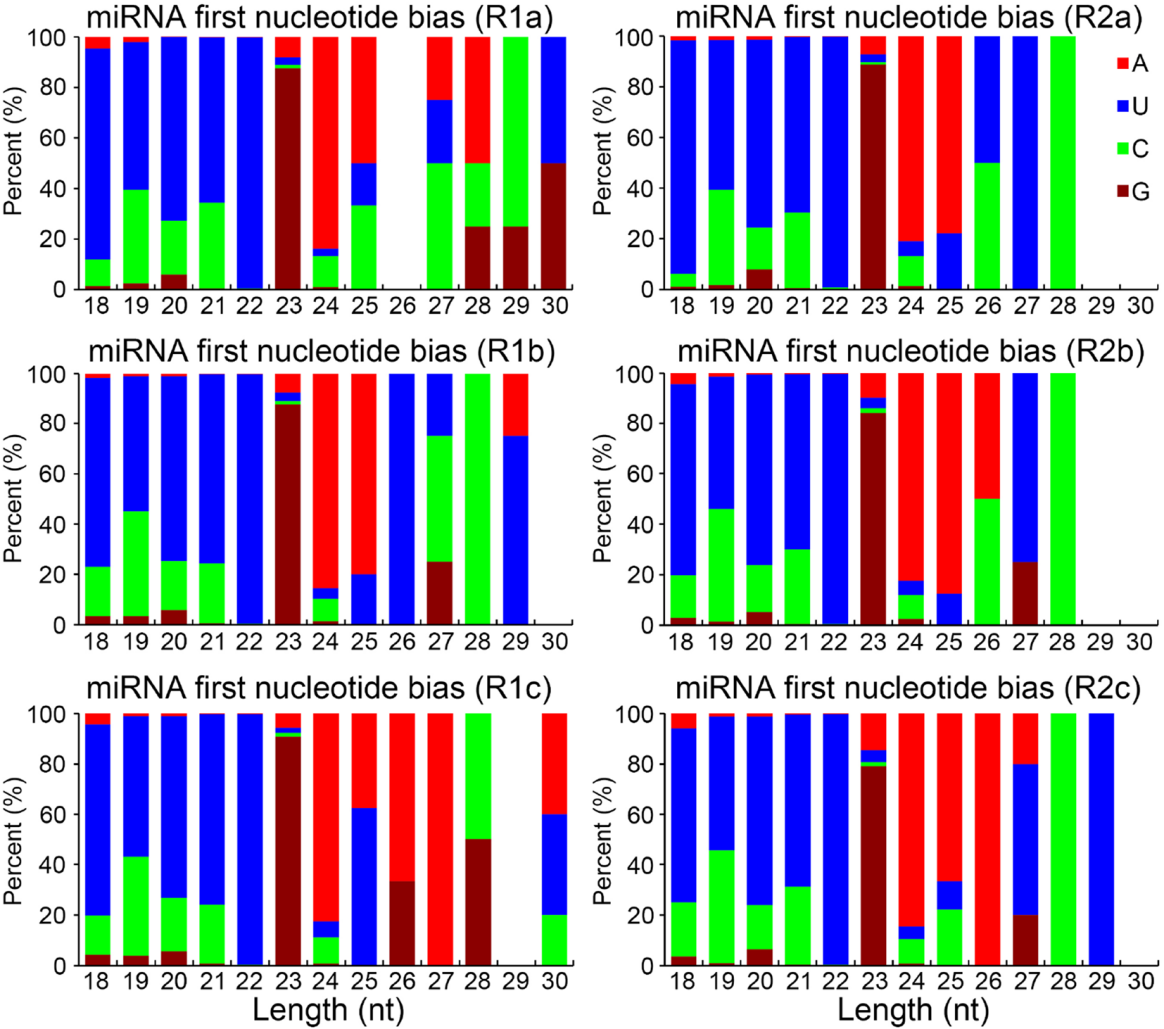
The preference to the first nucleotide of miRNAs according to the novel miRNA length.

To detect novel miRNAs, the unannotated reads of the six libraries were mapped onto the *A. bidentata* transcriptome. This resulted in the identification of 36 novel miRNAs in this plant species, of which 31 were detected in R1a, 34 in R1b, 34 in R1c, 33 in R2a, 33 in R2b and 34 in R2c (Tables S4, S5 of Supplementary Material 1). The predicted corresponding hairpin structures corresponding to these ranged 59–267 nt in length, their MFEIs from − 0.85 to − 1.91 (Table S5 of Supplementary Material 1). In all six libraries, nine of these miRNAs were classified as rare class, with a read count below 10. The length distribution of novel miRNAs along with the percentage of their read frequencies were looked into, with 21 nt and 22 nt RNAs being the most abundant (Fig. S1 of Supplementary Material 2). The frequency of occurrence of the first base of the novel miRNAs was also explored for all sRNA libraries, revealing that the 18–22 nt miRNAs preferentially started with “U” (> 60%), while 24 nt and 25 nt miRNAs mainly exhibited “A” as the first base (> 80% and > 60%, respectively) (Fig. 3). These 36 novel miRNAs, which, to the best of our knowledge, have not been identified in any other plant species to date, are considered to be novel or specific to *A. bidentata*. They have been assigned the names abi-miRn1-36 (Table S5 of Supplementary Material 1) and their secondary structures were shown in Supplementary Material 3.

### Screening, target prediction and functional analysis of differential expression (DE) miRNAs

**Figure 4:**
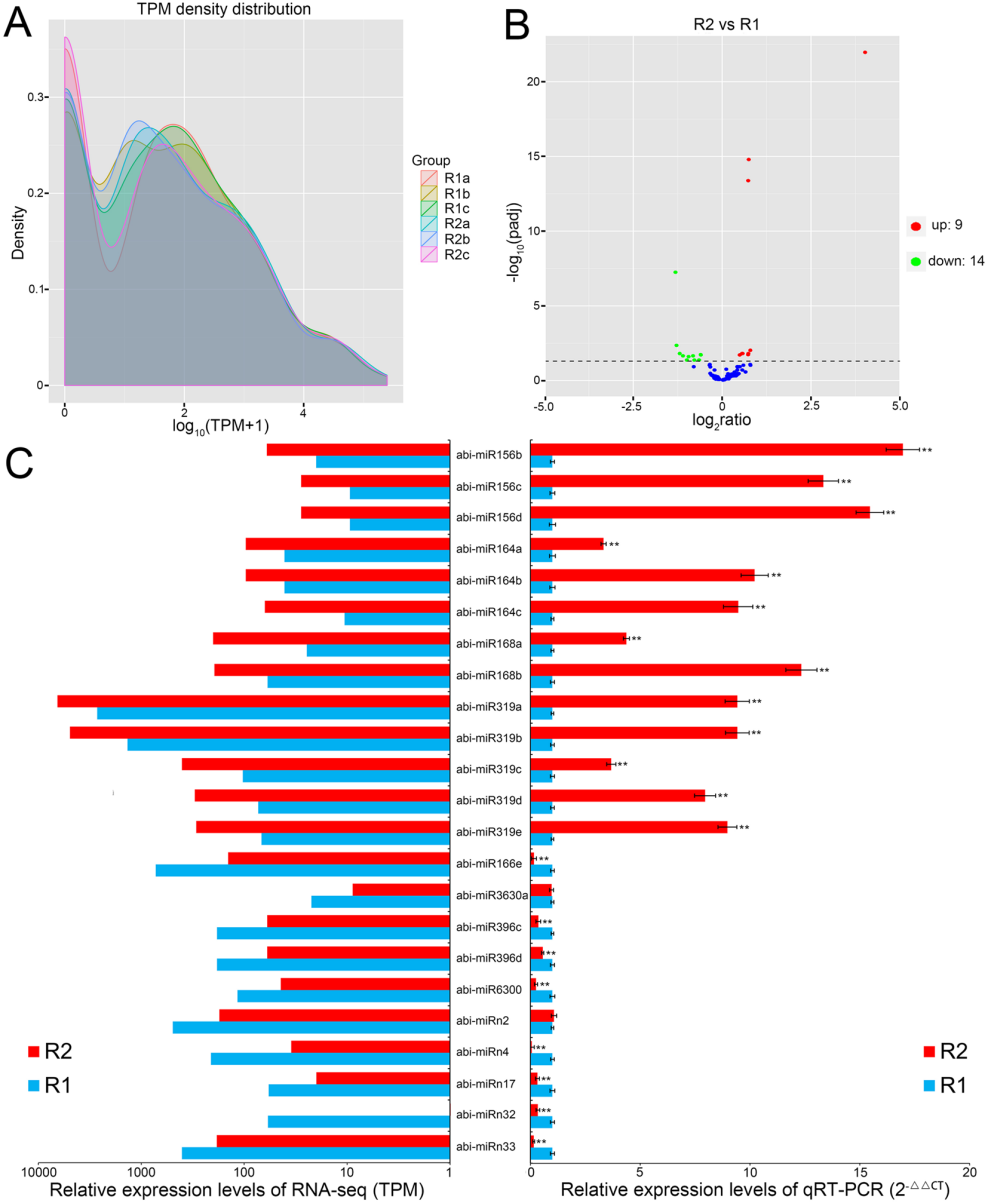
TPM density distribution (**A**), scatterplot (**B**), and RNA-seq and qRT-PCR analysis comparison (**C**) of DE miRNAs between R1 and R2 samples.

The expression abundance (transcripts per million reads, TPM) density distribution of novel and conserved miRNAs in the six libraries was shown in Fig. 4A. To screen DE miRNAs in response to the replanting benefit, nine novel and 151 conserved miRNAs for which the read count was < 10 were removed from each dataset; the read counts for the remaining 27 novel and 116 conserved miRNAs were normalized (Table S6 of Supplementary Material 1). A scatterplot illustrating the distribution of the DE miRNAs between R1 and R2 samples is depicted in Fig. 4B. We compared the abundance of normalized miRNAs between the R1 and R2 libraries, and the results identified the DE miRNAs in R1 and R2 (Table S6 of Supplementary Material 1). Among these miRNAs, 14 miRNAs were significantly up-regulated and nine were down-regulated in the R2 samples. Furthermore, of these DE miRNAs, miRn32 was only detected in R2. Quantitative real-time PCR (qRT-PCR) method was used to validate the differential abundance predicted by the RNA-seq analysis for the 23 DE miRNAs (Fig. 4C and Table S6 of Supplementary Material 1). The estimated abundance from the RNA-seq outcome and from qRT-PCR analysis was consistent for 21 of the 23 (the exceptions were abi-miR3630 and abi-miRn2) (Fig. 4C and Table S6 of Supplementary Material 1). Thus the 21 DE miRNAs-validated by qRT-PCR were further analyzed.

**Figure 5:**
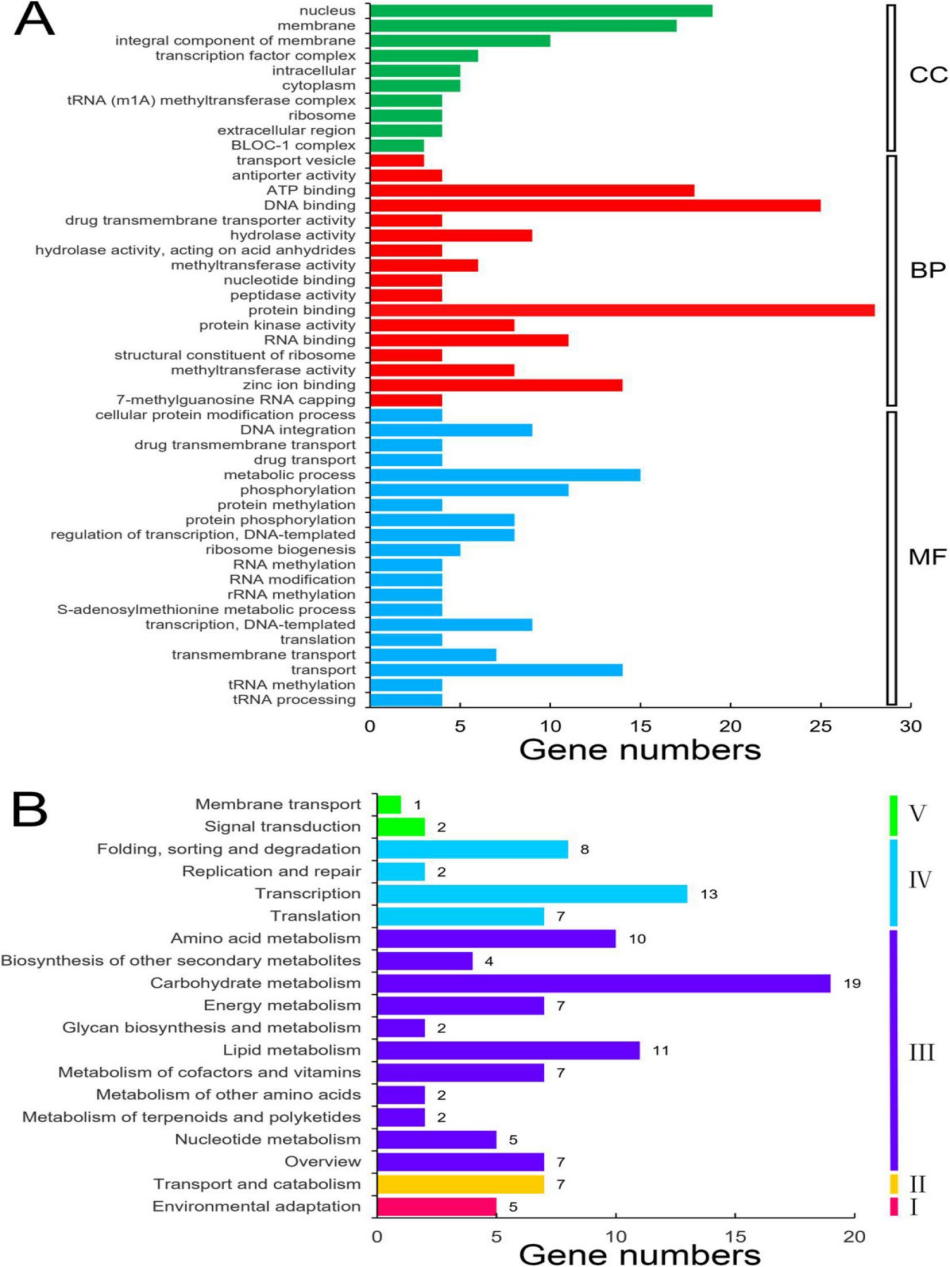
Gene Ontology (**A**) and KEGG (**B**) classifications of DE miRNA targets. *CC* cellular component, *BP* biological process, *MF* molecular function, *I* organismal systems, *II* cellular processes, *III* metabolism, *I* genetic information processing, *V* environmental information processing.

Based on the *A. bidentata* transcriptome data, we predicted a total of 169 target genes of the 21 DE miRNAs by considering only the common hits obtained between psRobot and psRNATarget programs (Table S7 of Supplementary Material 1). All 21 miRNAs were predicted to target more than one type of transcript. Functional annotation of these targets was performed by sequence comparison against the Nr/Nt, Pfam, Swissprot, KOG and GO databases, and 125 of the genes were annotated in at least one of these databases (Table S8 of Supplementary Material 1). The genes included fifteen squamosa promoter-binding-like genes (*SPL6* and *SPL*16) by abi-miR156 targets, three NAC domain-containing genes (*NAC1*) by abi-miR164 targets, four transcription factor bHLH74 genes (*bHLH74*) by abi-miR396, and thirteen transcription factor TCP genes (*TCP*) by abi-miR319 targets. Based on the GO analysis, 101 of the detected targets were organized into 47 functional groups (Fig. 5A). In the “cellular component” category, the predominant groups identified were “nucleus” and “membrane”; in the “molecular function” category, the most frequently identified groups were “protein binding” and “DNA binding”; in the “biological process” category, the predominant groups were “transport” and “metabolic process”. A search within the KEGG database revealed matches for 121 of the targets, these genes were assigned to 19 pathways of hierarchy 2 (groups classified based on pathway hierarchy 1) (Fig. 5B). The five identified categories based on pathway hierarchy 1 identified were “organismal systems” (5 genes), “cellular processes” (7 genes), “metabolism” (76 genes), “genetic information processing” (30 genes) and “environmental information processing” (3 genes). The most highly represented pathway was “carbohydrate metabolism” (19 genes) of the overall “metabolism” category.

### Integrated analysis of DE miRNA-target modules

**Figure 6:**
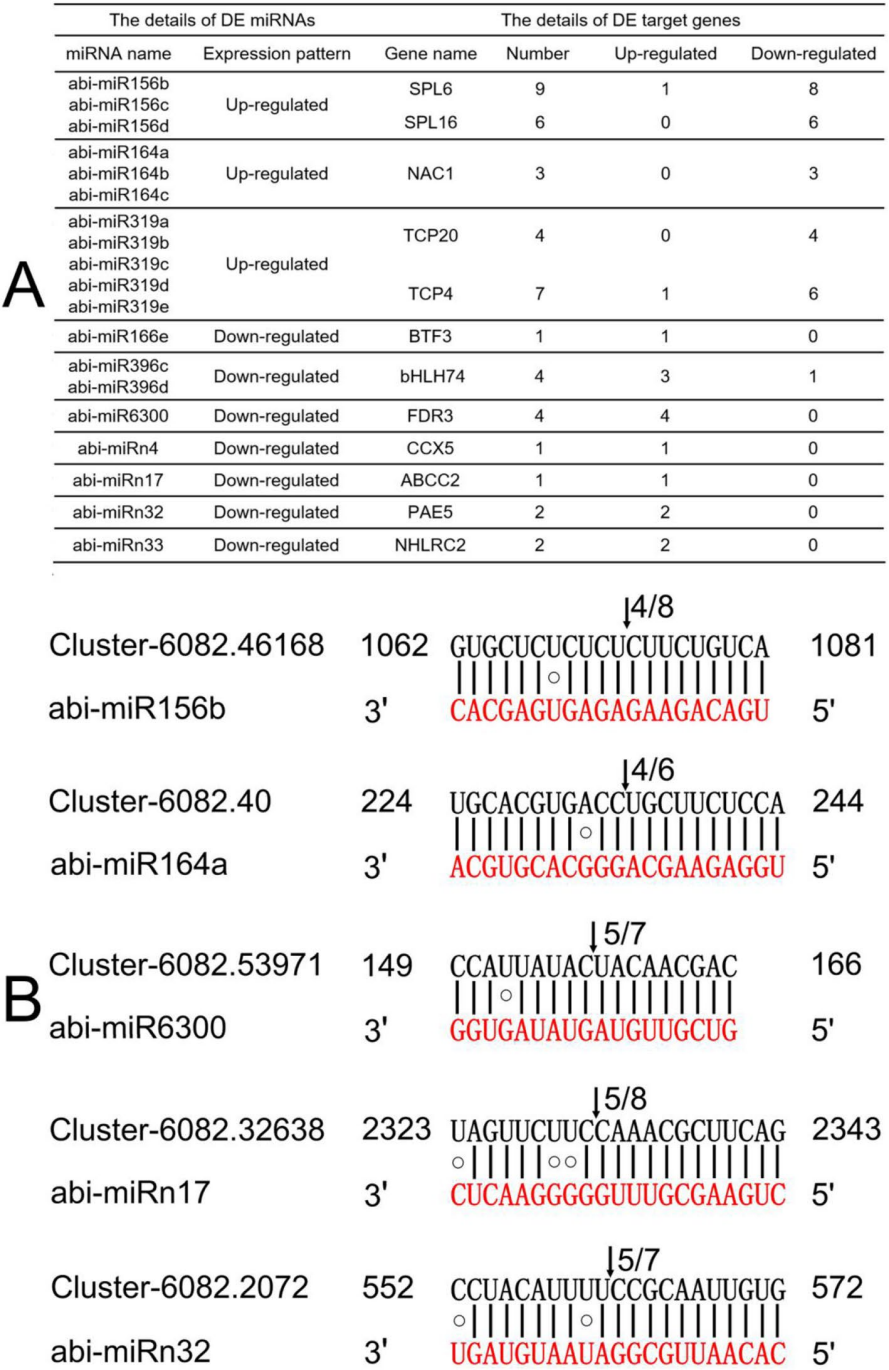
Summary and validation of the representative DE miRNA-target modules. (**A**) Summary of the modules; (**B**) RLM-RACE identification of 5 chosen cleavage events of the modules (Arrows point out the cleavage sites, and numbers represent the clones sequenced and how many were corrected).

To investigate the transcript abundance relations between the DE miRNAs and their corresponding target genes, the 125 annotated target gene expression profiles were analyzed based on R1 and R2 RNA-seq data (SRA accession number: SRP107934). As shown in Fig. 6A and Table S9 of Supplementary Material 1, 44 of the target genes that were significantly different expression in R1 and R2 roots. Among the DE targets, 29 genes corresponded to 11 up-regulated miRNAs and 15 genes corresponded to 8 down-regulated miRNAs. The results showed that the 10 miRNA-mRNA family modules may mostly indicate negative regulation (Fig. 6A), including 14 up-regulated genes (except one gene (Unigene ID: Cluster-6082.51459) encoding transcription factor bHLH74 (*bHLH74*)) and 27 down-regulated of the genes (except one (Unigene ID: Cluster-6082.52890) encoding squamosa promoter-binding-like protein 6 (*SPL6*) and one (Unigene ID: Cluster-18912.7) encoding transcription factor TCP4 (*TCP4*)).

To further verify the DE miRNA-target modules, five cleavage events were chosen for RNA ligase-mediated rapid amplification of cDNA ends (RLM-RACE) analysis (Fig. 6B). The results showed that one *SPL* (Cluster-6082.46168), one *NAC1* (Unigene ID: Cluster-6082.40) and one gene (Unigene ID: Cluster-6082.53971) encoding MATE efflux family protein FRD3 (*FDR3*) could be cleaved by three conserved miRNAs, abi-miR156b, abi-miR164a and abi-miR166e, respectively. Furthermore, we found that abi-miRn17 and abi-miRn33 as novel miRNAs could cleave one gene encoding ABC transporter C family member 2 (*ABCC2*; Unigene ID: Cluster-6082.32638) and one encoding NHL repeat-containing protein 2 (*NHLRC2*; Unigene ID: Cluster-19316.0), respectively.

### Validation of DE miRNA-target module expression patterns with qRT-PCR analysis

**Figure 7:**
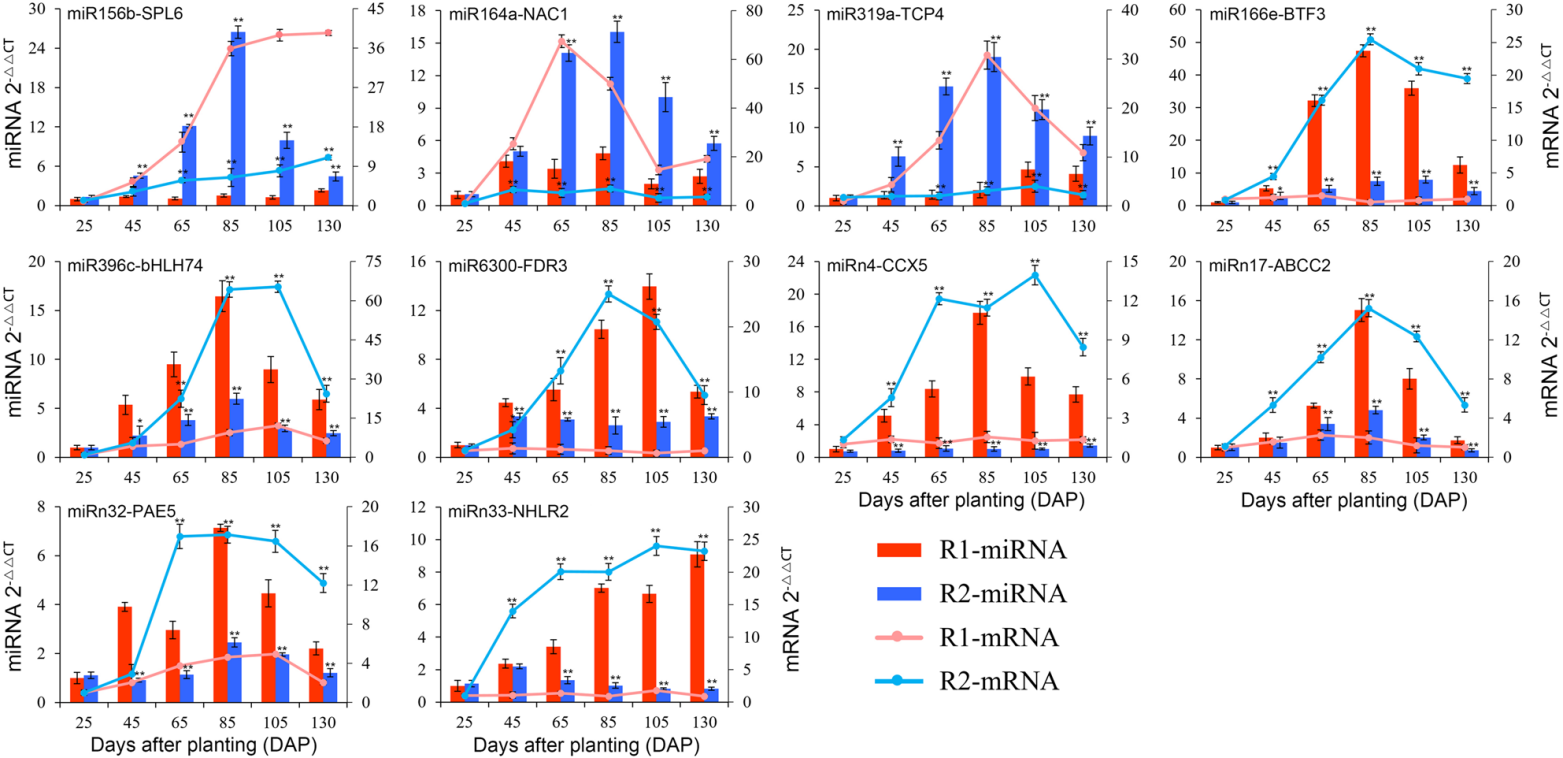
Expression patterns of 10 miRNA-target modules from R1 and R2 samples.

To validate the negative relationship between the expression patterns of the miRNAs and their key target genes, the transcript profiles of the selected 10 DE miRNA-target modules were assessed in the R1 and R2 roots from the various developmental stages. On the whole, the expression patterns of the selected mRNA targets were inversely related to those of the corresponding DE miRNAs at most of these stages (Fig. 7). For example, the expression pattern of abi-miR164a was significantly up-regulated in R2 samples from 65 to 130 DAP, whereas the pattern of *NAC1*, one abi-miR164a target gene, was significantly down-regulated in R2 at the corresponding stages; similarly, the expression pattern of abi-miR319a was significantly up-regulated in R2 samples from 45 to 130 DAP, whereas the pattern of *TCP4*, one abi-miR319a target gene, was down-regulated in the samples at the corresponding stages. Oppositely, a *bHLH74*, one target gene of down-regulated abi-miR396c, exhibited significantly higher expression in R2 than that in R1 from 65 to 130 DAP; similarly, a NHL repeat-containing protein 2 gene (*NHLRC2)*, one target gene of down-regulated abi-miRn33, exhibited significantly higher expression in R2 than that in R1 at four of the development stages.

## Discussion

### Transcriptome-wide identification of conserved and novel miRNAs

*A. bidentata* is a typical perennial medicinal herb with the replanting benefit because of its positively allelopathic effect under continuous monoculture conditions^5–7^. Although the previous study was initially conducted to explore the molecular mechanism of mRNA transcript regulation underlying the replanting benefit in *A. bidentata*^6^, the mechanism of mRNA post-transcript regulation was still not understood. miRNAs play important regulatory roles during the response of plants to various environments^12–16^. However, little is yet known regarding the spectrum of miRNAs present in *A. bidentata*, considering certain miRNAs may be responsible for the replanting benefit at the post-transcriptional level.

With the development of RNA-seq technology, researchers can easily obtain information on novel and conserved miRNAs from some species and their expression differences among various samples of a species^11,15,17^. Herein, we investigated the regulatory molecular regulator mechanism of the replanting benefit of *A. bidentata*, we had explored greatly to this number by RNA-seq platform to characterize the root miRNAome of R1 and R2 plants and obtaining > 85 million sRNA clean reads. The results of the present study revealed a range of sRNAs, of length 18–30 nt in length, containing substantially more of 24 nt sRNAs than of 21 nt, similar to the sRNA size distribution of sRNAs in several plant species, including *A. thaliana*, broccoli and ramie^17,19,20^. These data suggested that the sequencing results were reliably representative of the endogenous sRNAs in *A. bidentata*.

The transcriptome of a given organism could provide valuable information for the identification of low-abundance or tissue-specific miRNAs and their targets^17,21^. In addition, the choice to use the reference transcriptome of *A. bidentata* for miRNA identification has been dictated by the lack of the complete genome for this species^22,23^. As such, we did not expect to be able to obtain a full representation of all miRNA loci existing in *A. bidentata*. Therefore, the sRNA sequences were instead aligned against mature plant miRNAs in miRBase, and 45 conserved miRNA families (including 267 miRNA members) in *A. bidentata* are reported in our study for the first time. A conservative analysis revealed that the majority of these conserved miRNAs in *A. bidentata* were known already reported in more than two other plant species^14,15,18^, implying that these sequences were very confident*.* In order to discover novel miRNAs of *A. bidentata*, we also carried out a stringent identification of the putative miRNA precursors through a series of filtering steps. In particular, applying the criterion of an MFEI threshold of − 0.85 for MFEI provided a set of well-resolved miRNA candidates^24,25^, supporting the reliability of the detection. Thus, the identification of these novel and conserved miRNAs in *A. bidentata* could provide an important basis for deciphering the miRNA-mediated molecular mechanism of the organism’s response to the replanting benefit and other environment stimuli.

### Possible miRNA role in the regulation of *A. bidentata* replanting benefit

**Figure 8:**
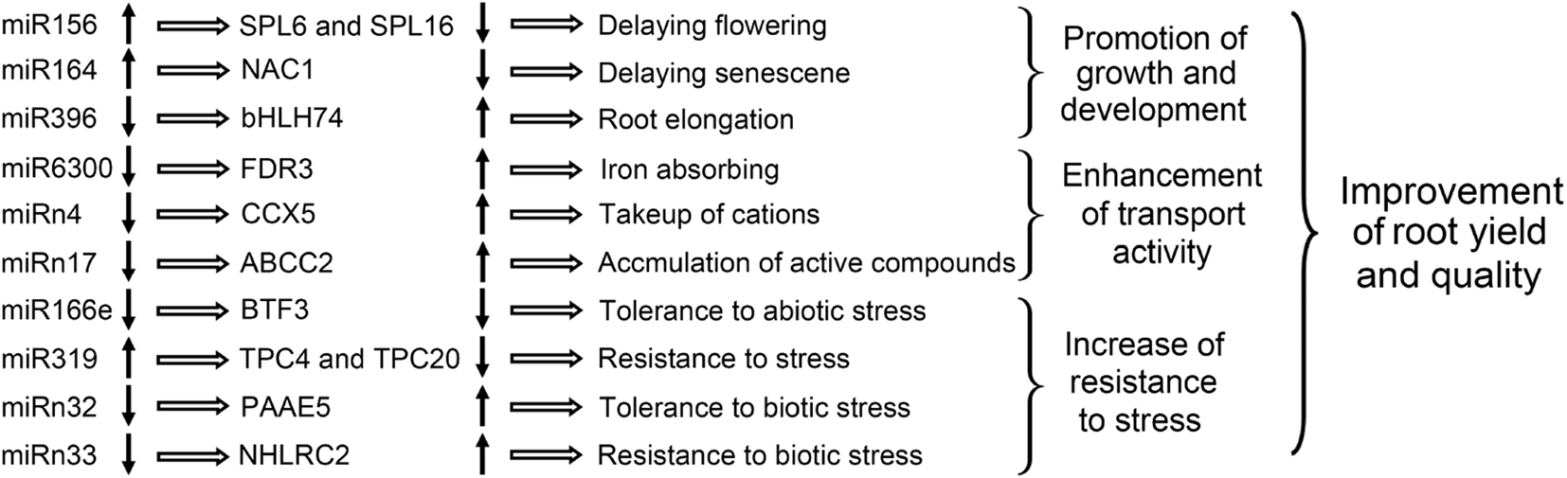
A hypothetical regulatory mechanism of DE miRNA-target modules involved in the replanting benefit of *A. bidentata* roots. ↑: upregulation of expression; ↓: downregulation of expression.

It has been documented that continuous monoculture leads to a pronounced increase in the yield and quality of *A. bidentata* roots^5–7^, a phenomenon that was reproduced in present study. In order to disclose the regulatory miRNA regulator roles involved in the replanting benefit, we identified a set of 21 DE miRNAs in R1 and R2 roots by RNA-seq and qRT-PCR technologies. Plant miRNAs function mainly as post-transcriptional gene regulatory molecules, mediating mRNA degradation by interacting with their complementary targets^10,13–17^. For exploring the potential functions of these miRNAs, we also predicted and partly validated their cleavage mRNA targets based on *A. bidentata* transcriptome sequences. The potential function and integrated expression pattern analysis of the key DE miRNA-targets supports the hypothesis that continuous monoculture of *A. bidentata* may reprogram the expression patterns of miRNA-target modules. A hypothetical regulatory mechanism of the key miRNA-target modules in the replanting benefit is described in detail below (Fig. 8).

Continuous monoculture of *A. bidentata* promotes root growth and development, exhibiting the thicker and longer of roots^5–7^. Here, several DE miRNA-target modules may be directly or indirectly involved in root growth and development. For example, up-regulated abi-miR156 in R2 was found to target two *SPLs*, which, when overexpressed in *A. thaliana*, resulted in the production of an early flowering phenotype^26,27^. Furthermore, in alfalfa, miR156 overexpression increased root regeneration capacity, resulting in plants with increased vegetative yield, delayed flowering, indicating that miR156-*SPL* modules serve a role in plant root growth^27,28^. It is therefore possible that, in replanted *A. bidentata*, a higher expression of the abi-miR156 represses the expression of *SPLs*, leading to delayed flowering and promoting root growth and development. Similarly, three up-regulated abi-miR164 members were found to target *NAC1*, which promotes *A. thaliana* and maize root development^29,30^. In strawberry, a significant increase in miR164 expression resulted in decreased *NAC1* expression and delayed its senescence under conditions of low temperatures^31^. Here, higher-expression miR164 in replanted *A. bidentata* may repress expression of *NAC1*, possibly delaying plant senescence time and promoting root development. By contrast, *bHLH74*, a target of abi-miR396 members, regulates root growth in *A. thaliana* by affecting the elongation zone in root development^32,33^. In replanted *A. bidentata*, down-regulated abi-miR396 targeted a *bHLH74*, implying that the upregulation of *bHLH74* may promote root elongation.

Replanted *A. bidentata* exhibits stronger root activity with an increase in productivity and quality^5,6^. This may be due to the regulatory of the several miRNA-target modules, which could enhance root transport activity, promoting the absorption of nutrients from the soil into the root and the accumulation of active compounds in roots. For example, abi-miR6300 targets a gene encoding MATE efflux family protein FRD3 (*FRD3*), which mediates the efflux of citrate into the root vasculature and is necessary for efficient iron transport in plants^34–36^. Overexpression of *FRD3* results in enhancing absorption of iron absorbed into the root cells, promoting root growth and increasing productivity^34–36^. Iron is a necessary, but often limiting, nutrient for plant growth and development^37^. Here, down-regulated abi-miR6300 increased the expression of *FRD3*, likely enhancing the absorption of iron from the soil into the roots. Similarly, one of abi-miRn4 targets is a cation/calcium exchanger 5 gene (*CCX5*), which is linked to the uptake of cation nutrients and the homeostasis of cellular Ca^2+^^38–40^. In replanted *A. bidentata*, down-regulated abi-miRn4 allowed the upregulation of *CCX5*, strengthening cation absorption into the root cells and balancing cellular Ca^2+^ levels. In addition, the content of active compounds is an important quality index of *A. bidentata* root^7^. A ABC transporter C family member 2 (*ABCC2*), one of the targets of abi-miRn17, which mediates the vacuolar transport and accumulation of endogenous active compounds including anthocyanins, flavonoid and folates^41–43^. In replanted *A. bidentata* roots, down-regulated abi-miRn17 allowed the enhancement of *ABCC2* expression probably promoting the transport and accumulation of some active compounds.

*A. bidentata* tolerance to biotic and abiotic stresses is strengthened under continuous monoculture conditions^5,6^. Here, we speculated that several miRNA-target modules may be involved in the resistance to various stresses. For example, abi-miR166e targeted one gene encoding transcription factor *BTF3*, which functions as a key regulator in the tolerance to adversity stress^44–46^. In tobacco and *A. thaliana, BTF3* increased the tolerance to abiotic stress^46,47^. Here, down-regulated abi-miR166e in replanted *A. bidentata* permitted the upregulation of *BTF3*, possibly strengthening the plant tolerance to various stresses. Similarly, it is well established that the activated miR319-*TCP* module is involved in stress resistance^48–52^. Overexpression of miR319 in rice, enhanced its tolerance to cold temperatures^51^, and in tomato plants, it enhanced the resistance to the root-knot nematode^52^. Here, upregulation of abi-miR319 repressed the transcription actor *TCP4* (as one of its targets) expression, likely strengthening the tolerance of replanted *A. bidentata* to various stresses. Moreover, abi-miRn32 targeted a pectin acetylesterase 5 gene (*PAE5*), which is up-regulated in the infection process of the root-knot nematode in *A. thaliana*^53^. In replanted *A. bidentata*, specific expression of abi-miRn32 could repress *PAE5* transcription, possibly strengthening the resistance to biotic stress. In addition, abi-miRn33 targeted a *NHLRC2*, whose upregulation in tomato is correlated with the increase of the resistance to pathogenic bacteria^54^. Here, down-regulated abi-miRn33 in replanted *A. bidentata* may promote *NHLRC2* expression, possibly strengthening the tolerance to biotic stress.

Overall, a clear effect of the replanting benefit of *A. bidentata* is the alteration in the miRNA content of the roots. The differential expression of the key miRNA-target modules in this plant might promote its root growth and development, enhance its transport activity of nutrients and active compounds, and strengthen its tolerance to various stresses, leading to the improvement in yield and quality. Whether the identified DE miRNAs actually regulate key targets responsible for these benefits requires further experimental demonstration. The identification of these miRNA-target modules has nevertheless succeeded in providing leads for determining the molecular genetic basis of the replanting benefit in *A. bidentata*.

## Materials and methods

### Cultivation and sampling of *A. bidentate*

The experiment was based on a single cultivar of *A. bidentata* (*Radix Achyranthis Bidentatae* “Number 1”). The R1 (as control) and R2 (treatment) crops were grown in pot-culture conditions from June 15 to September 30, 2019. As R1 samples, the plants were grown in the fresh soil from a field where no *A. bidentata* had been planted for at least 20 years, while R2 samples, the plants were replanted in the soil from the field in which the cultivar had been planted in the previous year. Three biological replicates of root samples were harvested from five plants at six developmental stages: The seedling stage 25 DAP, the branching stage (45 DAP), the flowering stage (65 DAP), the seed-setting stage (85 DAP), physiological maturity (105 DAP) and final harvest (130 DAP). The samples were used for morphological characteristics measure and miRNA-mRNA patterns using quantitative real-time PCR (qRT-PCR), while the 85 DAP samples only were used for constructing of sRNA-seq libraries and validating DE miRNA profiles via qRT-PCR method.

### Assay of root morphological characteristics and biomass

The root samples from various stages were cleaned to remove the soils for evaluating their morphological characteristics, and then these roots were baked to become dry for assessing their dry weight. Each examination was preformed with three biological replicates.

### RNA isolation, quantification and qualification

Total RNA from each sample was isolated using the TRIzol reagent (Invitrogen, Carlsbad, CA, USA) according to the manufacturer’s instructions, then treated with RNase-free DNase I (Qiagen, Hilden, Germany) to remove contaminating genomic DNA. RNA purity was measured by the Nanodrop 2000 instrument (Thermo Scientific, Wilmington, DE, USA), its concentration assessed using the Qubit RNA Assay Kit (Life Technologies, CA, USA) and its integrity estimated using the RNA Nano 6000 Assay Kit (Agilent Technologies, CA, USA) with RIN number over 7.0.

### sRNA-seq library construction

The sRNA libraries were constructed using 1 μg of total RNA according to the instruction of the NEBNext Multiplex Small RNA Library Prep Set for Illumina (NEB, USA). The process is as follows: firstly, the total RNA was ligated with 3′ RNA adapter, and followed by 5′ adapter ligation. Secondly, reverse transcription followed by PCR was performed to create cDNA constructs based on the sRNAs ligated with 3′ and 5′ adapters. Thirdly, small cDNA fractions that range from 140 to 160 bp (the length of sRNA plus the 3′ and 5′ adaptors) were isolated by using a 8% polyacrylamide gel electrophoresis (100 V, 80 min), recovered and dissolved in 8 μL elution buffer. Fourthly, the cDNA library quality was assessed on the Agilent Bioanalyzer 2100 system using DNA High Sensitivity Chips. At last, the libraries were sequenced using Illumina Hiseq 2500 platform, and 50 bp single-end reads were generated. Clean reads were obtained for removing low quality reads containing ploy-N, with 5′ adapter contaminants, without 3′ adapter or the insert tag, containing ploy A or T or G or C and other low quality reads from raw data. At the same time, Q20, Q30, and GC-content of the raw data were calculated. Then, remained 18–30 nt reads were perform the downstream analysis.

### Identification and analysis of conserved miRNAs

The clean reads were mapped to the *A. bidentata* transcriptome sequences^6^ from NCBI SRA database (https://www.ncbi.nlm.nih.gov/sra) using Bowtie^55^ without mismatches in order to analyze their expression level and distribution on the reference transcripts. We then removed the sequences that could be mapped to non-coding RNAs (tRNA, rRNA, snRNA and snoRNA sequences) by performing alignments with the Rfam 14.0 (http://rfam.xfam.org), Noncode 5.0 (http://www.noncode.org) and Silva (http://www.arb-silva.de) databases using the SOAP2 tool^56^. To identify conserved miRNAs and analyze their conservation, the filtered reads were mapped using BlastX searches against plants’ mature miRNAs from the miRBase 22.1 database (http://www.mirbase.org) with no mismatches and a threshold of 1 × 10^−3^ E-value^57^.

### Identification and analysis of novel miRNAs

For novel miRNA identification, the remaining unannotated 18–25 nt sRNAs in length were be mapped onto the *A. bidentata* transcriptome^6^. The characteristics of miRNA precursor (pre-miRNA) hairpin structures can be used to predict novel miRNAs by miREvo^58^ and miRDeep-P^59^ software packages were integrated to predict novel miRNAs through the exploration of secondary structure. Only sRNA sequences that fit the following the criteria were designated as potential miRNAs^60^: the pre-miRNA folded into a perfect stem-loop hairpin structure; the potential miRNA sequences located on one arm of hairpin structure; the loops or breaks were not allowed in the miRNA/miRNA* duplex; no more than 6 nt mismatches were allowed between miRNA/miRNA* duplex; the percentage of total content of A and U bases ranged from 30 to 75%; and the pre-miRNA had a high negative minimal free-folding energy and MFE index (MFEI > 0.85) which [(MFE/length of the sRNA sequence) × 100]/(G + C)%^24,25^.

### Abundance and differential expression analysis of miRNAs and mRNA genes

miRNA expression levels were calculated as TPM as follows^61^: For the samples with biological replicates, DE analysis between the R1 and R2 (R2/R1) groups was performed using the DESeq R package (v1.8.3)^62^. The resulting P-values were adjusted using a previously established method for controlling the false discovery rate (FDR < 0.001)^63^. A adjusted P-value (padj) of 0.01 with the |log_2_ratio|> 1 was set as the default threshold below which miRNAs were considered to be significantly DE. For mRNA samples, the DE levels between R1 and R2 samples were assessed as per kilobase of transcript sequence per millions base pairs sequenced (FPKM) using the above mentioned transcriptome data^6^, the padj, the FDR and |log_2_ratio| values of the default threshold below which mRNA were considered to be significantly DE^6^.

### Prediction and functional analysis of miRNA targets

miRNA targets were predicted using psRobot (http://omicslab.genetics.ac.cn/psRobot) and psRNATarget (http://plantgrn.noble.org/psRNATarget) tools with default parameters^64,65^. The targets were annotated against the Nr/Nt (http://www.ncbi.nlm.nih.gov), Pfam (http://pfam.xfam.org), Swissprot (http://www.uniprot.org) and KOG (http://www.ncbi.nlm.nih.gov/COG) databases, using the BlastN algorithm and a threshold E-value of 1 × 10^−5^. Gene ontology (GO, http://sraww.geneontology.org) terms were obtained using the Blast2GO software^66^. Pathway assignment was carried out by means of the KEGG database^67^ and removed the “Human Diseases” category in the KEGG pathway hierarchy 1.

### RNA ligase-mediated rapid amplification of cDNA ends (RLM-RACE)

RLM-RACE was performed according to the instruction of the GeneRacer kit (Invitrogen, USA). Briefly, total RNA (5 μg) from roots was first removed the 5′ phosphates with calf intestinal phosphatase (CIP) and ligated to GeneRacer RNA Oligo adapter. The cDNA was then transcribed using GeneRacer Oligo dT primers. RACE-PCR was performed with using 5′ universal primers (Invitrogen, USA) and 3′ gene-specific primers (designed by Oligo 7.0 and synthesized by Sangon, Shanghai, China), respectively (Table S10 of Supplementary Material 1). The nested PCR products were purified with the TaKaRa MiniBEST Agarose Gel DNA Extraction Kit and subcloned into the pMD-18 vector (Takara, Tokyo, Japan), which was then used to transform *E. coli*. At least eight independent clones of each constructed vector were verified by sequencing (Sangon, Shanghai, China).

### QRT-PCR analysis of miRNA or mRNA genes

In order to validate the transcript abundance of miRNAs or mRNA genes between R1 and R2 samples, the relative expression levels of miRNAs or mRNA genes were analyzed by qRT-PCR on a QuantStudio 6 Flex qRT-PCR system (Applied Biosystems, Foster City, CA, USA). The miRNAs specific, stem-loop RT primers and the *18S rRNA* (Genebank ID: LT992958.1) gene primers were supplied by miRNA Design V1.01 software (Vazyme, Nanjing, China). Gene specific primers for mRNA genes and the *Actin* (Genebank ID: JZ970405) gene were designed by Beacon Designer 8.0 software (Premier Biosoft International, Palo Alto, CA, USA), with estimated melt temperature of 55–60 °C and amplification length of 90–150 bp. The primer sequences are listed in Table S11 and S12 of Supplementary Material 1.

For miRNA qRT-PCR analysis, 50 ng total RNA was reverse transcribed with a MiRNA 1st strand cDNA Synthesis Kit (by stem-loop) (Vazyme, Nanjing, China) using miRNA stem-loop RT primer. Reverse transcription was carried out in 20 μL reaction, which was incubated at 25 °C for 5 min, 50 °C for 15 min and 85 °C for 5 min. qRT-PCR was performed with a miRNA Universal SYBR qPCR Master Mix Kit (Vazyme, Nanjing, China) in 20 μL reaction mixtures containing 1 μL cDNA, 10 μL 2× miRNA Universal SYBR qPCR Master Mix, 1 μL miRNA specific primer, 1 μL mQ Primer R (Reverse universal primer) and 7 μL nuclease-free water.

For mRNA qRT-PCR analysis, 2 µg total RNA was reverse-transcribed in a 20 µL reaction containing 5U M-MLV reverse transcriptase (Takara, Tokyo, Japan) according to the manufacturer’s instructions. Each 25 µL reaction contained 0.2 μM of each primer, 12.5 µL SYBR Premix EX Taq (Takara, Tokyo, Japan) and 100 ng cDNA. All reactions including the sRNA and cDNA samples, no-template controls were run, and the reference genes as internal controls were also amplified. The qPCR protocol was as follows: 95 °C for 30 s, followed by 36 cycles of 95 °C for 10 s, 56–60 °C (Tables S11, S12 of Supplementary Material 1) 30 s and 72 °C for 30 s and a final ramping of 60–95 °C to determine the amplico’s dissociation behavior. Three biological replicates were included per sample. Three technical replicates for each biological replicate were used. The relative expression levels of the miRNAs or mRNA genes were calculated using the 2^−ΔΔCT^ method^68^ and the data were normalized on the basis of the reference genes *18S rRNA* and *Actin*, respectively. For statistical analysis, significant differences of the data were used to Student’s *t-*test method (P < 0.05 or 0.01) using SPSS22.0 software.

## Supplementary Information

Supplementary Information 1.

Supplementary Information 2.

Supplementary Information 3.
