# Supplementary material for: Integrated miRNA-mRNA analysis reveals the roles of miRNAs in the replanting benefit of Achyranthes bidentata roots
Source: Sci Rep. 2021 Jan 15;11:1628. doi: 10.1038/s41598-021-81277-6 (PMC7810699; doi:10.1038/s41598-021-81277-6)

**Integrated miRNA-mRNA analysis reveals the roles of miRNAs in the replanting benefit of *Achyranthes bidentata* roots**

Yan Hui Yang^1^*, Ming Jie Li^2^, Yan Jie Yi^1^, Rui Fang Li^1^, Cui Xiang Li^1^, Heng Yang^1^, Jing Wang^1^, Jing Xuan Zhou^1^, Sui Shang^1^, Zhong Yi Zhang^2^*

# ^1^College of Bioengineering, Henan University of Technology, Lianhua Street 100, Zhengzhou High-technology Zero, Henan Province, China, 450001

# ^2^College of Crop Sciences, Fujian Agriculture and Forestry University, Jinshan Road, Cangshan District, Fuzhou, China, 350002

# *Corresponding author e-mail: [yyhui2004@126.com](mailto:yyhui2004@126.com);

# Tel: +86-371-67756928

# Fax: +86-371-67756928

**Supplementary material 2**

Fig. S1 The distribution of reads along with novel miRNA length.


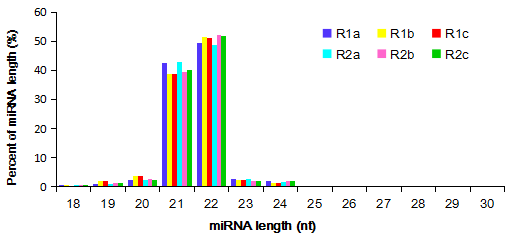

Supplement: Supplementary file 2 — Supplementary Information 2. [file 41598_2021_81277_MOESM2_ESM.doc]
