# Supplementary material for: Integrated miRNA-mRNA analysis reveals the roles of miRNAs in the replanting benefit of Achyranthes bidentata roots
Source: Sci Rep. 2021 Jan 15;11:1628. doi: 10.1038/s41598-021-81277-6 (PMC7810699; doi:10.1038/s41598-021-81277-6)

**Integrated miRNA-mRNA analysis reveals the roles of miRNAs in the replanting benefit of *Achyranthes bidentata* roots**

Yan Hui Yang^1^*, Ming Jie Li^2^, Yan Jie Yi^1^, Rui Fang Li^1^, Cui Xiang Li^1^, Heng Yang^1^, Jing Wang^1^, Jing Xuan Zhou^1^, Sui Shang^1^, Zhong Yi Zhang^2^*

# ^1^College of Bioengineering, Henan University of Technology, Lianhua Street 100, Zhengzhou High-technology Zero, Henan Province, China, 450001

# ^2^College of Crop Sciences, Fujian Agriculture and Forestry University, Jinshan Road, Cangshan District, Fuzhou, China, 350002

# *Corresponding author e-mail: [yyhui2004@126.com](mailto:yyhui2004@126.com); zyzhang@fafu.edu.cn

# Tel: +86-371-67756928

Fax: +86-371-67756928

**Supplementary material 3** Secondary structure of novel abi-miRNA precursors

1. Secondary structure of abi-miRn1 precursor


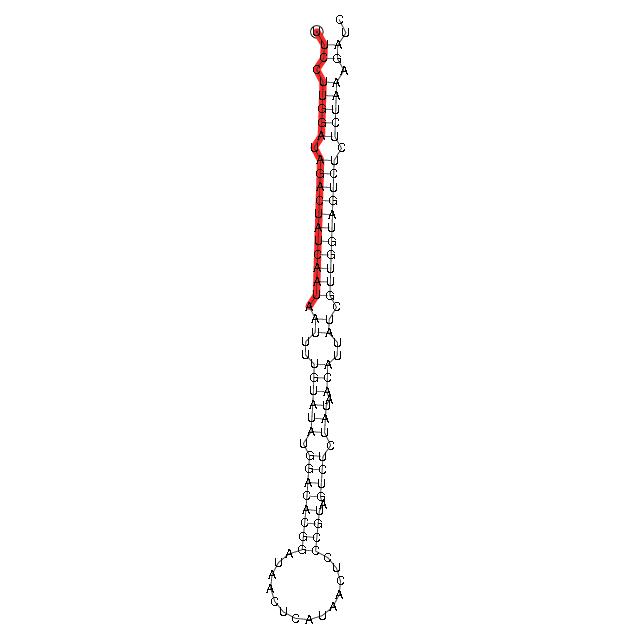


1. Secondary structure of abi-miRn2 precursor


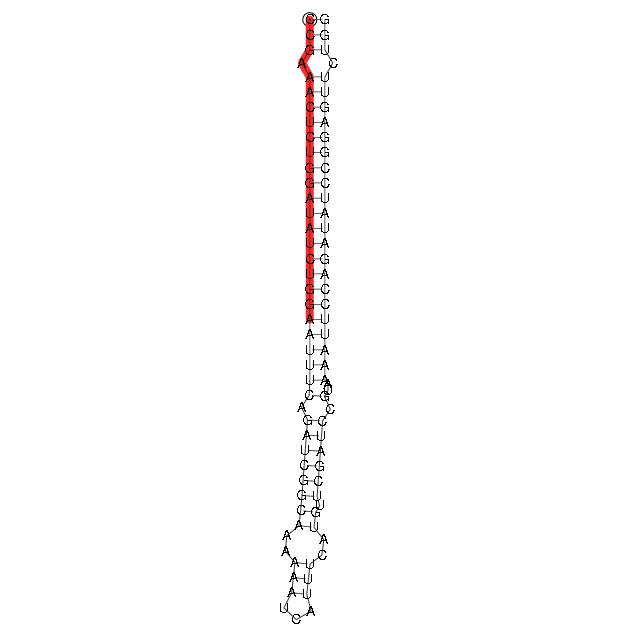


1. Secondary structure of abi-miRn3 precursor


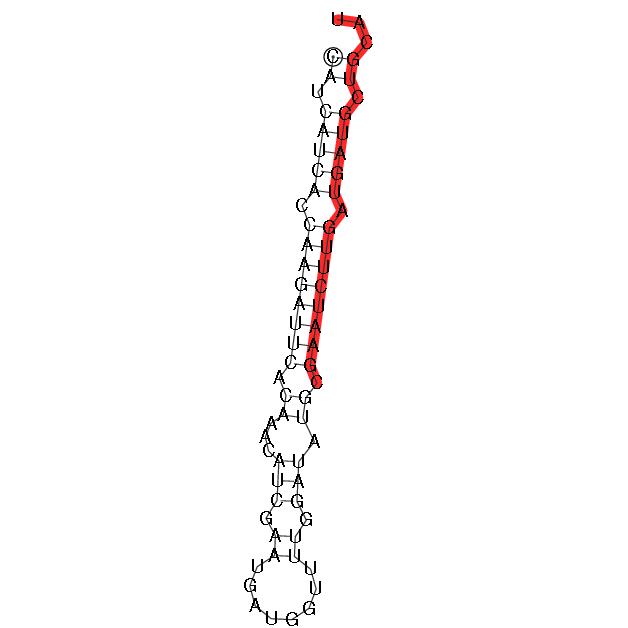


1. Secondary structure of abi-miRn4 precursor
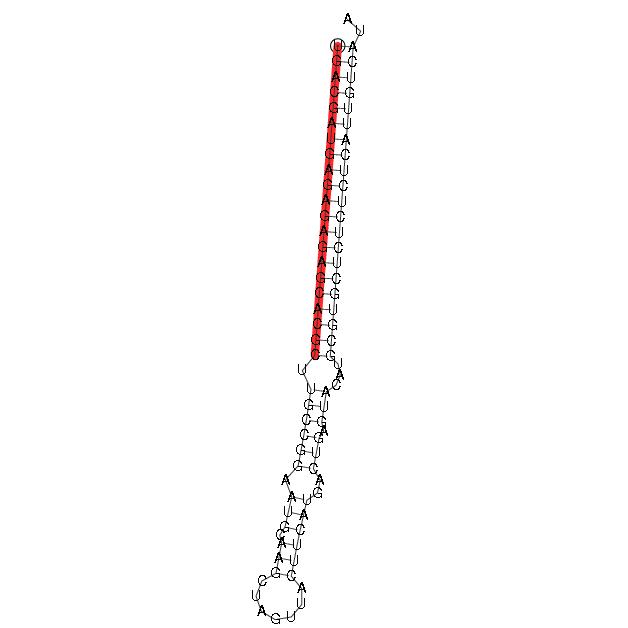

2. Secondary structure of abi-miRn5 precursor


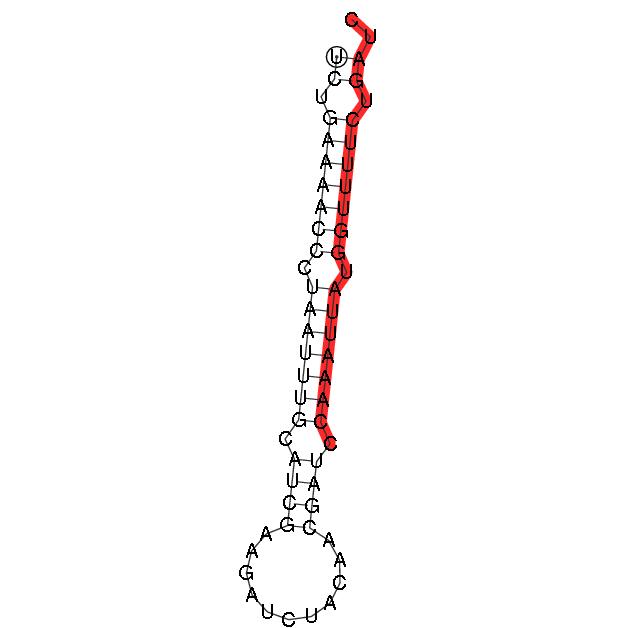


1. Secondary structure of abi-miRn6 precursor


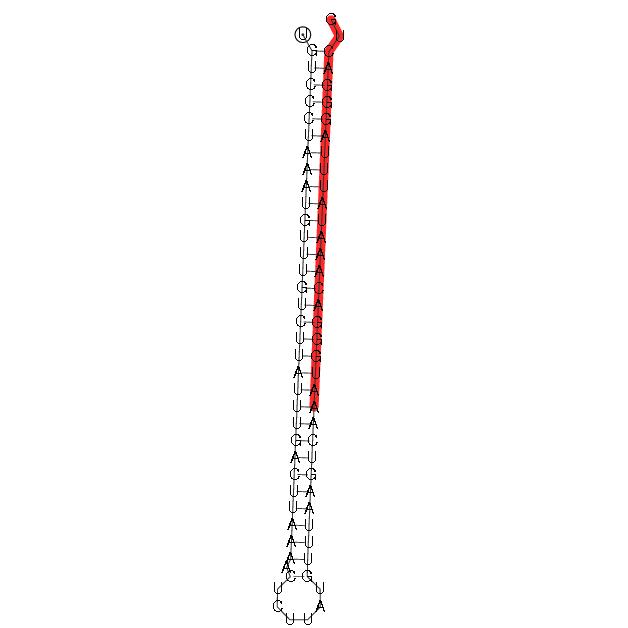


1. Secondary structure of abi-miRn7 precursor


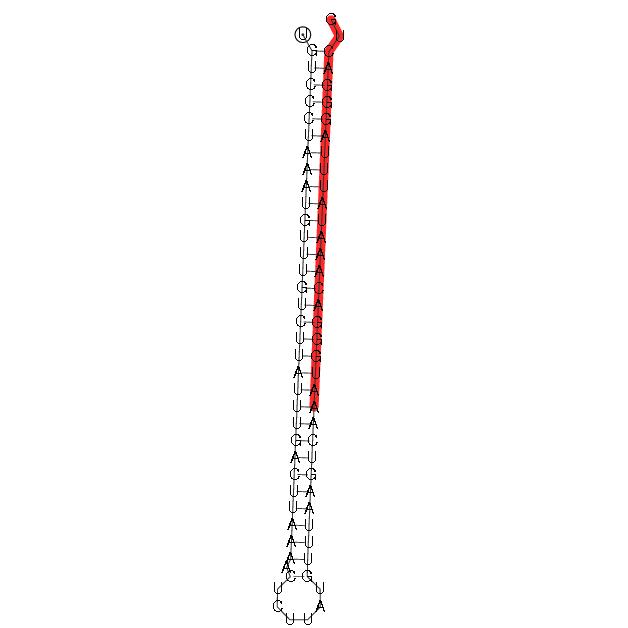


1. Secondary structure of abi-miRn8 precursor


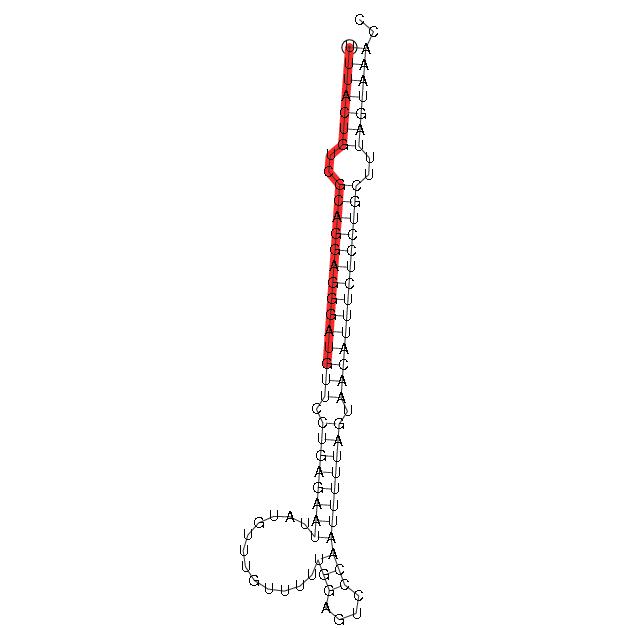


1. Secondary structure of abi-miRn9 precursor


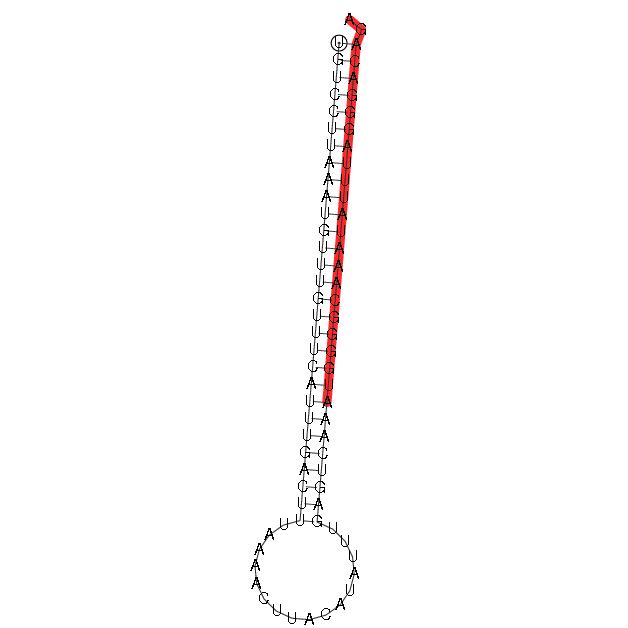


1. Secondary structure of abi-miRn10


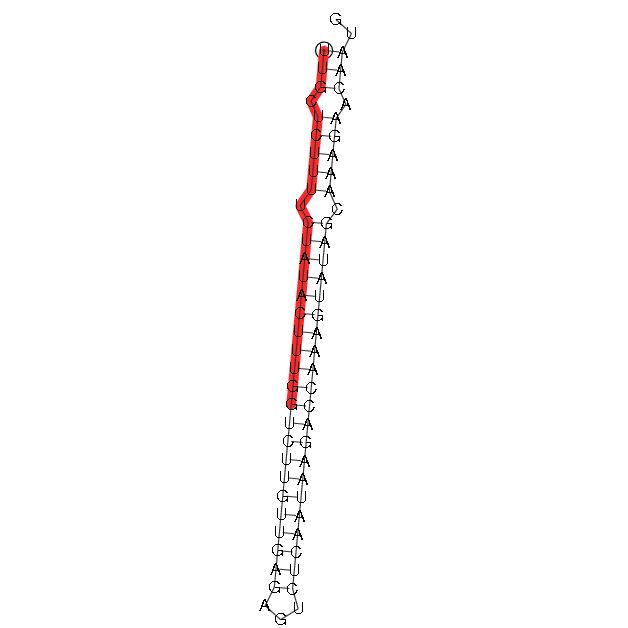


1. Secondary structure of abi-miRn11 precursor


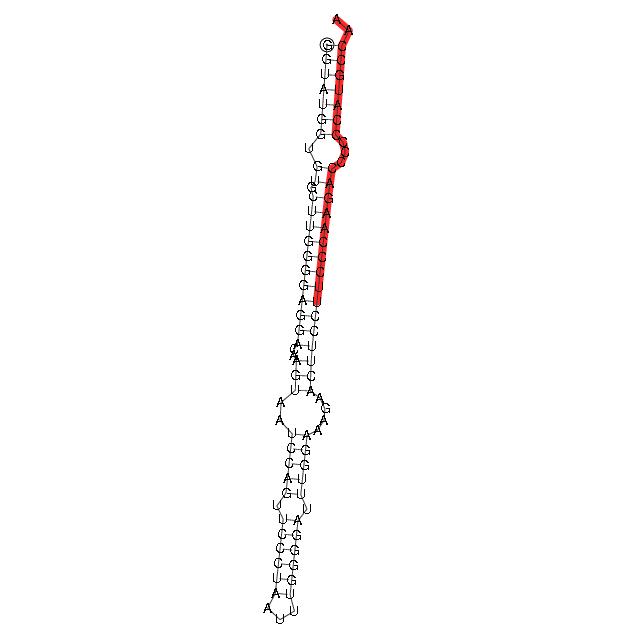


1. Secondary structure of abi-miRn12 precursor


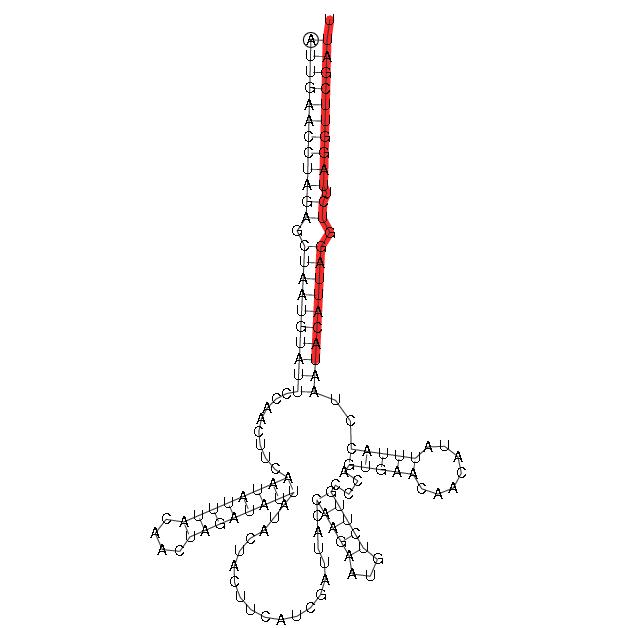


1. Secondary structure of abi-miRn13 precursor


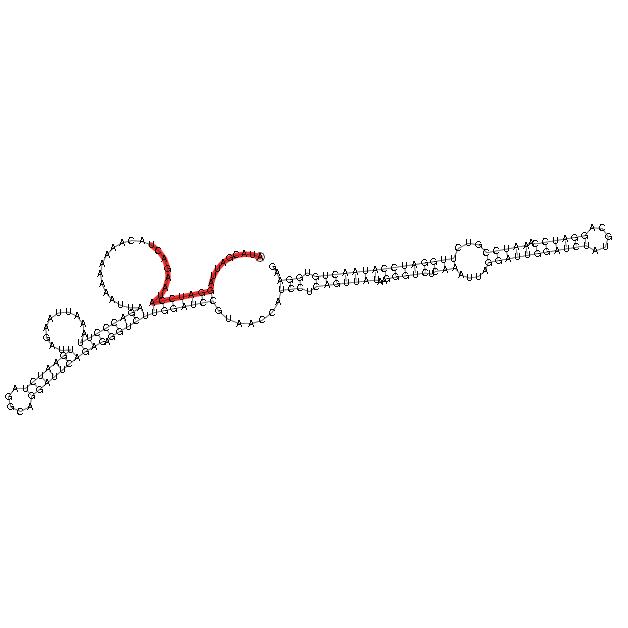


1. Secondary structure of abi-miRn14 precursor


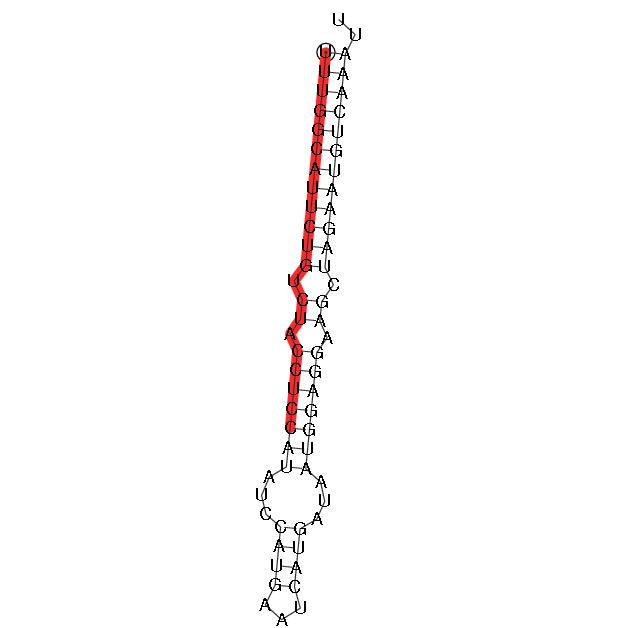


1. Secondary structure of abi-miRn15 precursor


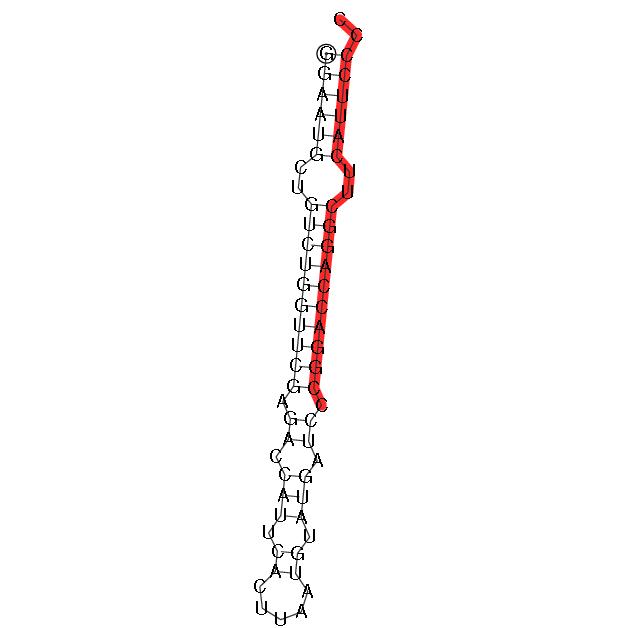


1. Secondary structure of abi-miRn16 precursor


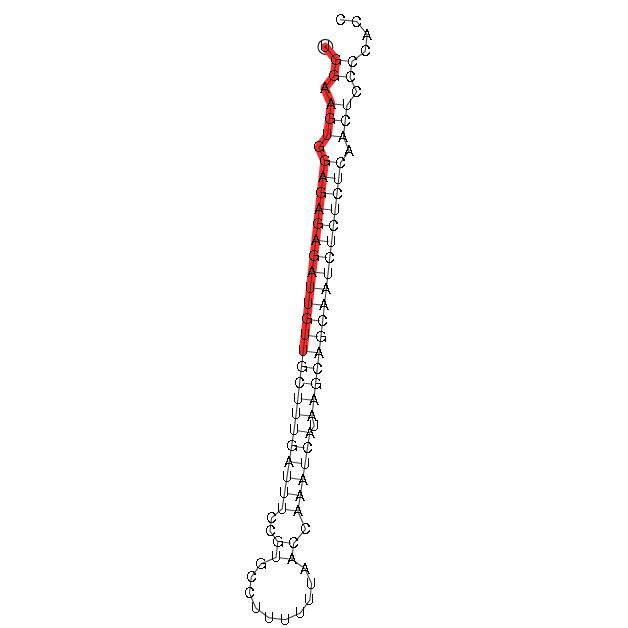


1. Secondary structure of abi-miRn17 precursor


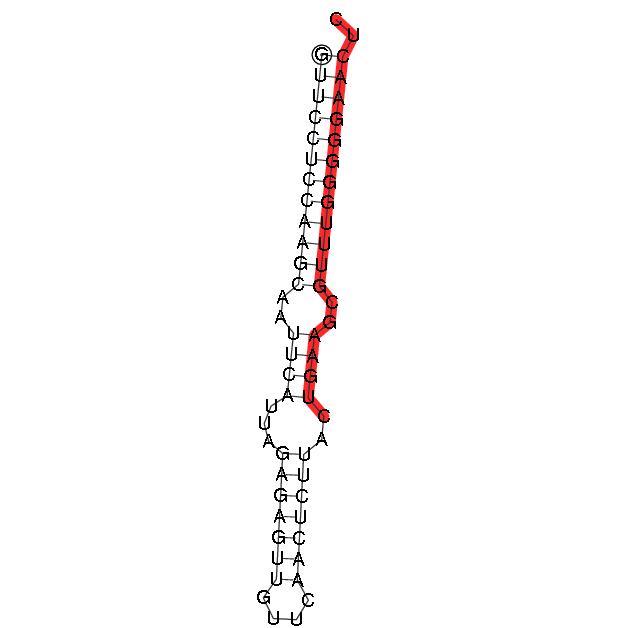


1. Secondary structure of abi-miRn18


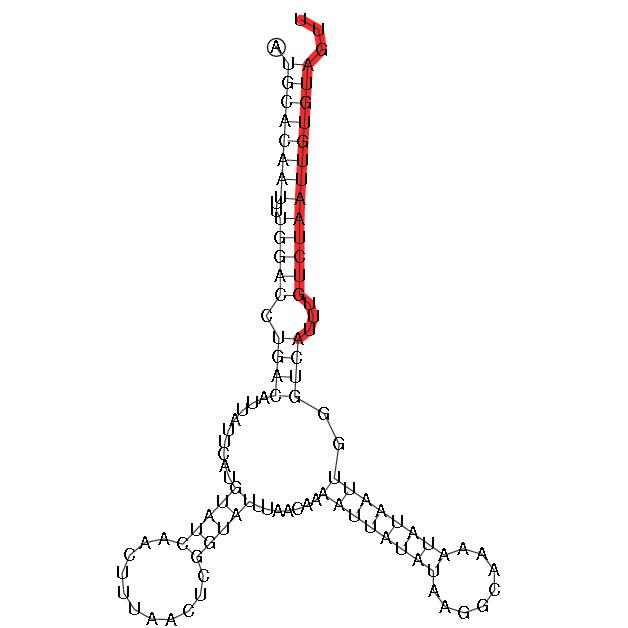


1. Secondary structure of abi-miRn19 precursor


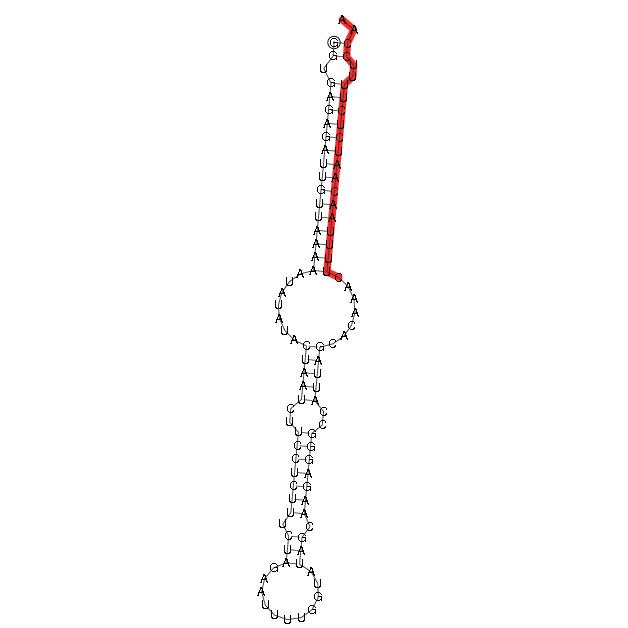


1. Secondary structure of abi-miRn20 precursor


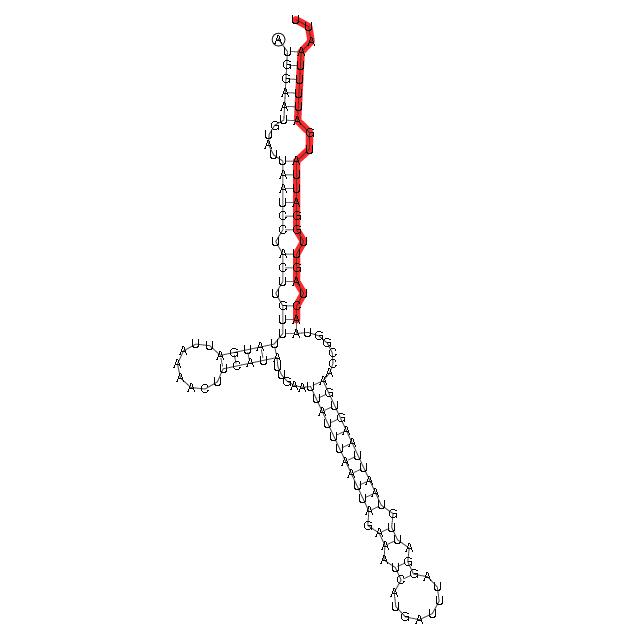


1. Secondary structure of abi-miRn21 precursor


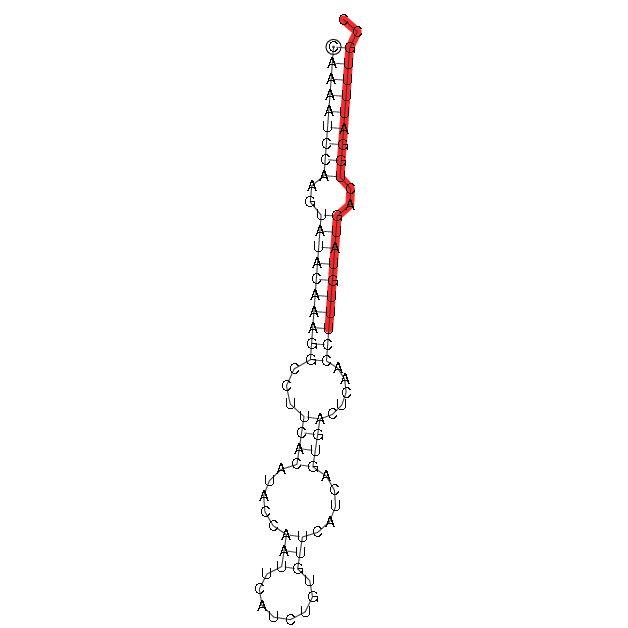


1. Secondary structure of abi-miRn22 precursor


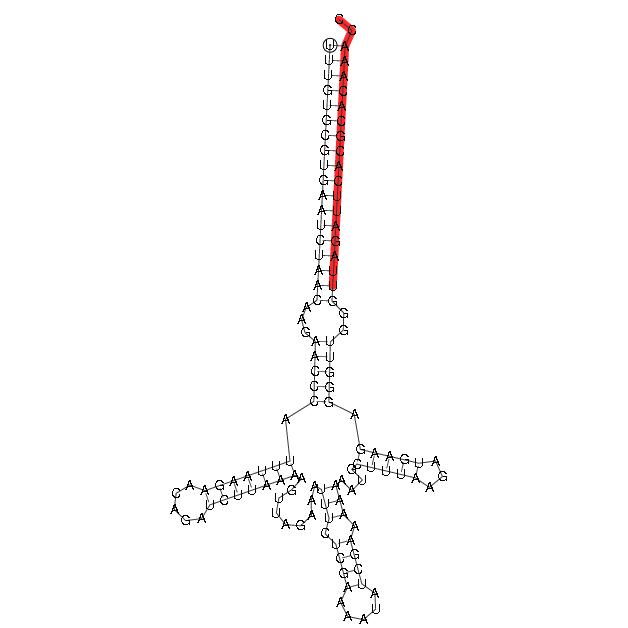


1. Secondary structure of abi-miRn23 precursor


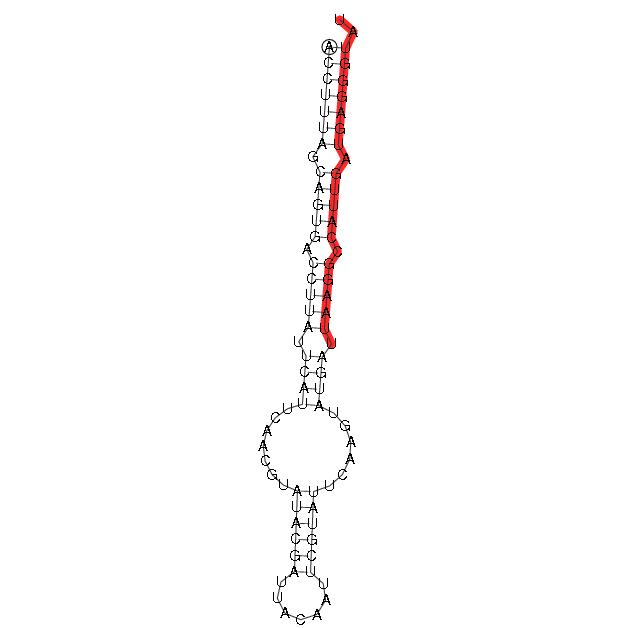


1. Secondary structure of abi-miRn24 precursor


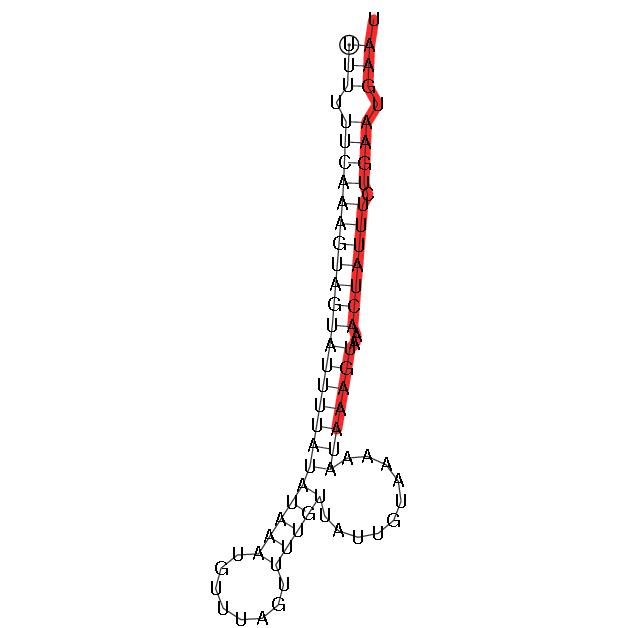


1. Secondary structure of abi-miRn25 precursor


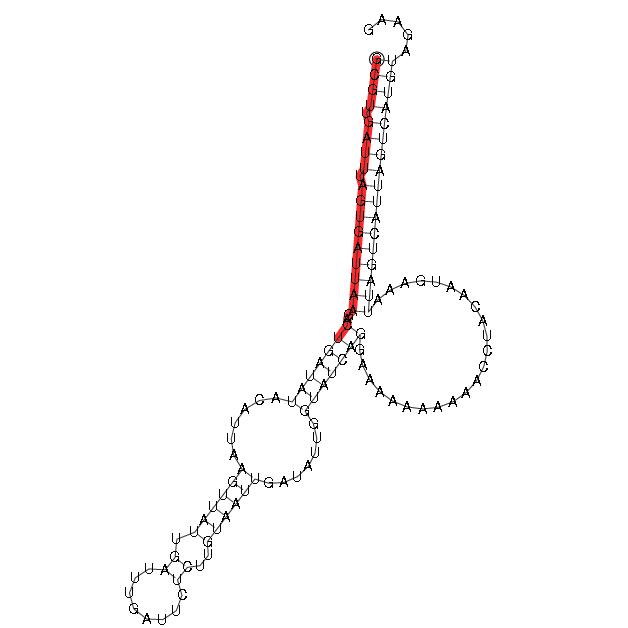


1. Secondary structure of abi-miRn26 precursor


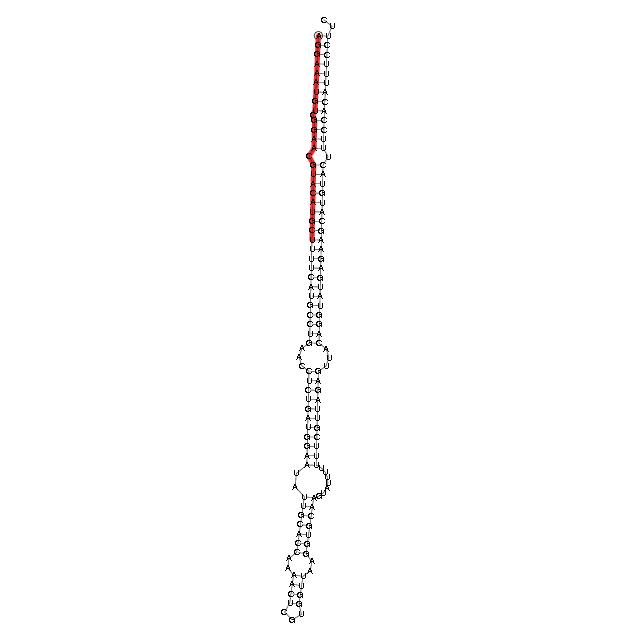


1. Secondary structure of abi-miRn27 precursor


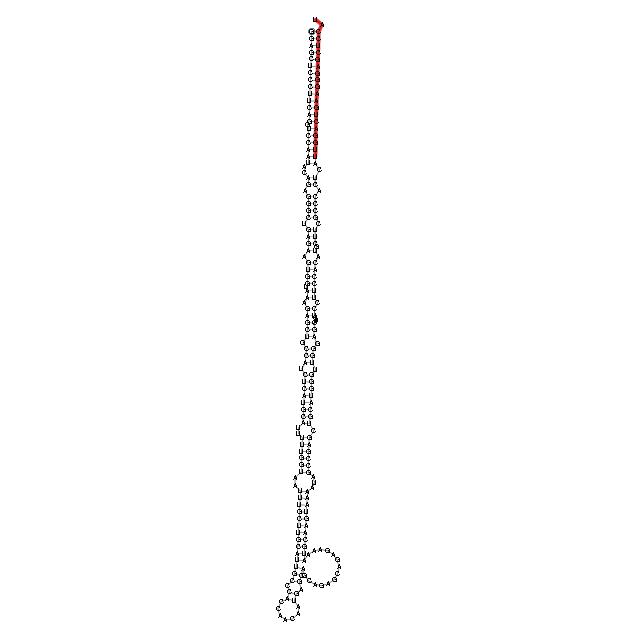


1. Secondary structure of abi-miRn28 precursor


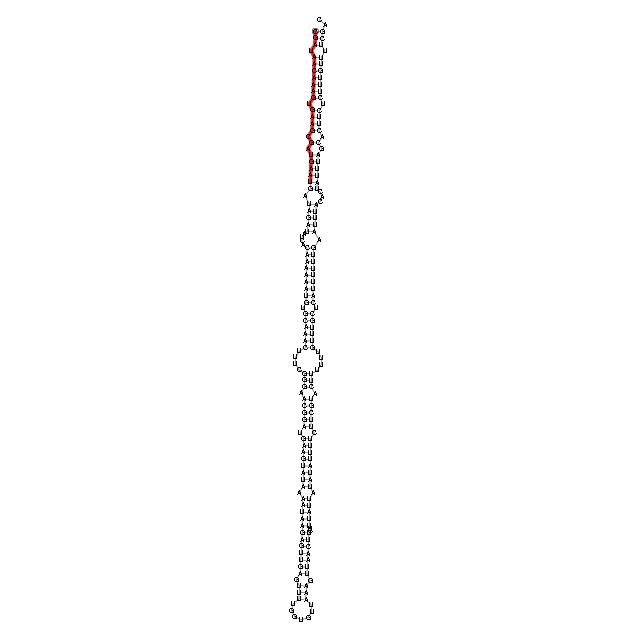


1. Secondary structure of abi-miRn29 precursor


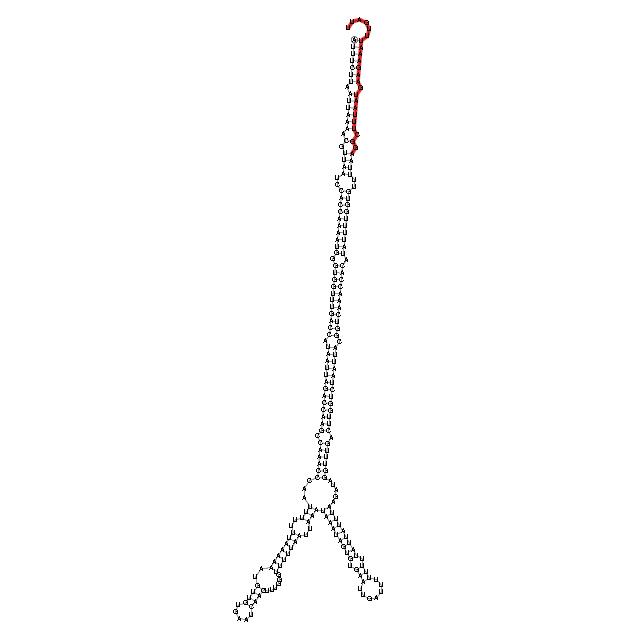


1. Secondary structure of abi-miRn30 precursor


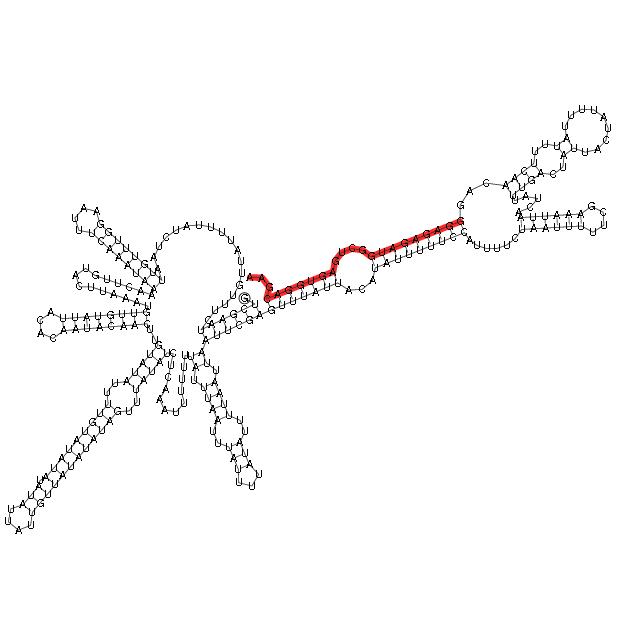


1. Secondary structure of abi-miRn31


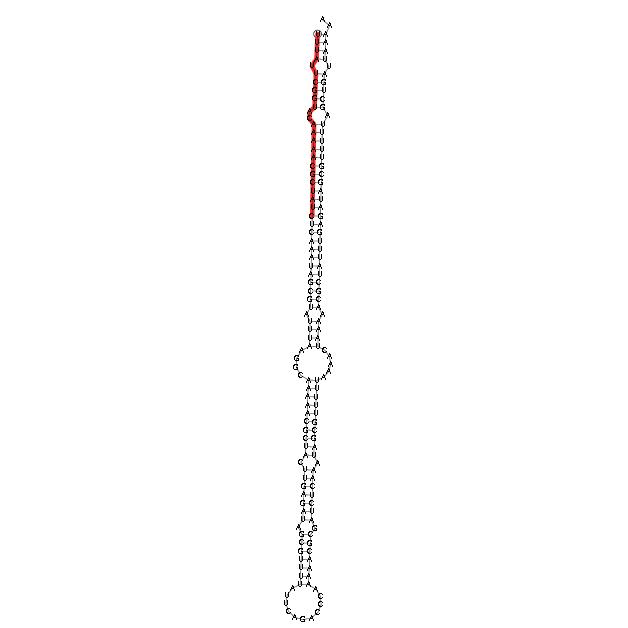


1. Secondary structure of abi-miRn32 precursor


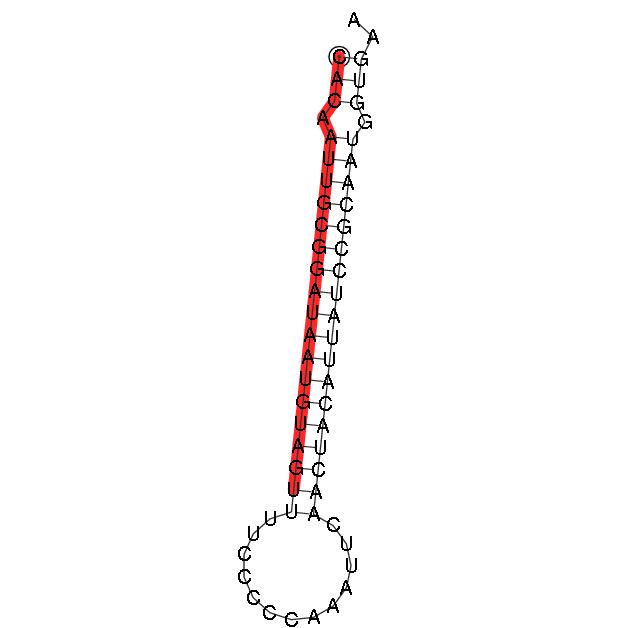


1. Secondary structure of abi-miRn33 precursor


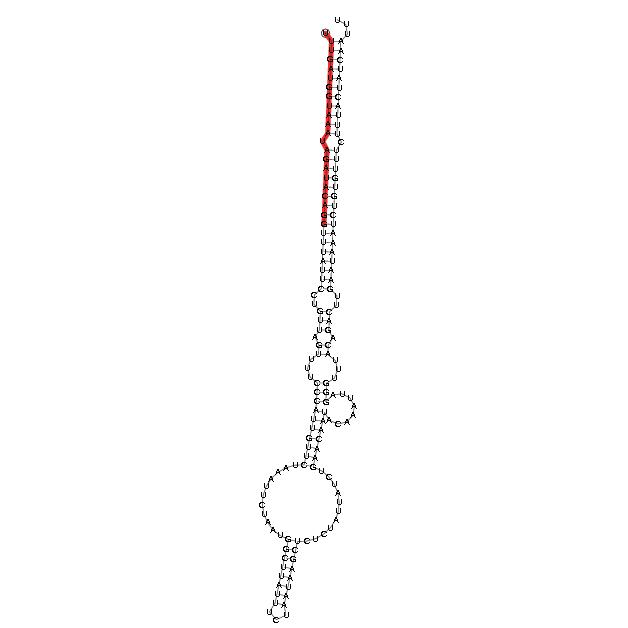


1. Secondary structure of abi-miRn34 precursor


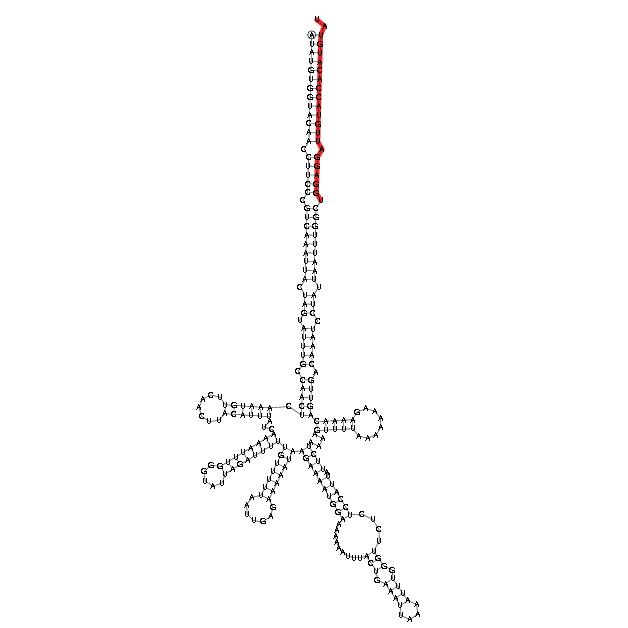


1. Secondary structure of abi-miRn35 precursor


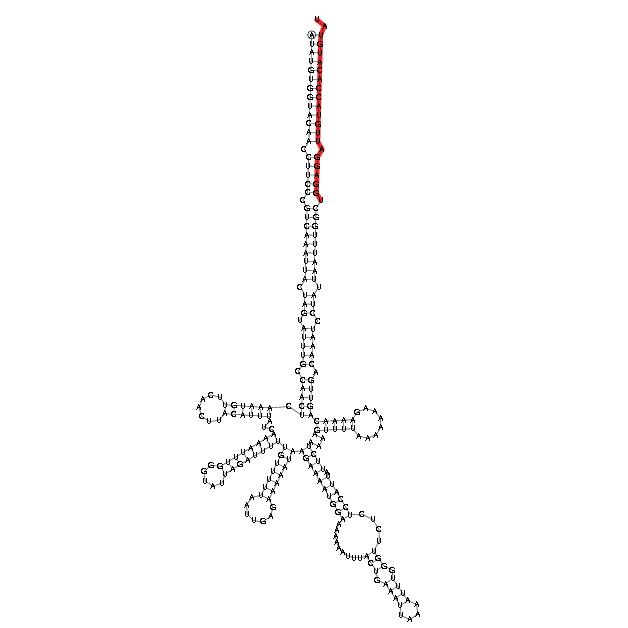


1. Secondary structure of abi-miRn36 precursor


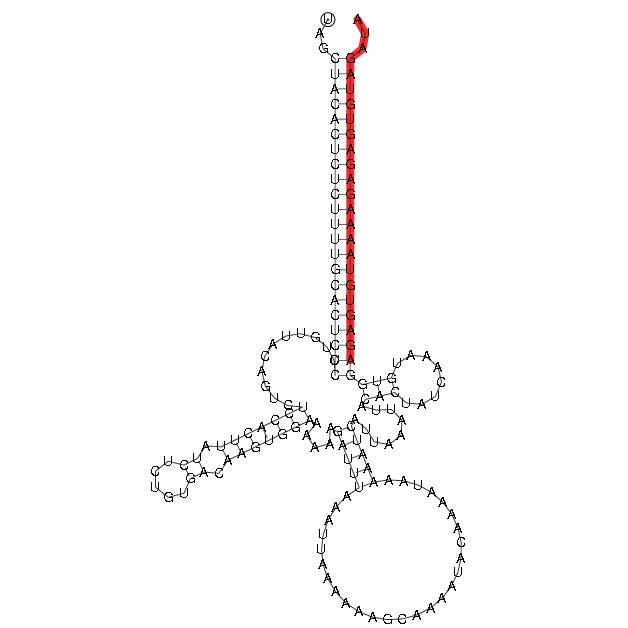

Supplement: Supplementary file 3 — Supplementary Information 3. [file 41598_2021_81277_MOESM3_ESM.docx]
